# Polysiloxane‐Based Single‐Ion Conducting Polymer Electrolyte for Sodium Batteries

**DOI:** 10.1002/advs.202600077

**Published:** 2026-07-14

**Authors:** Yixuan Guo, Hyokyeong Kang, Maider Zarrabeitia, Ludovica Roselli, Hyerim Kim, Vittorio Marangon, Jang‐Yeon Hwang, Dominic Bresser

**Affiliations:** ^1^ Helmholtz Institute Ulm (HIU) Ulm Germany; ^2^ Karlsruhe Institute of Technology (KIT) Karlsruhe Germany; ^3^ Department of Energy Engineering Hanyang University Seoul Republic of Korea; ^4^ Department of Battery Engineering Hanyang University Seoul Republic of Korea; ^5^ Ulm University (UUlm) Ulm Germany

**Keywords:** battery, polymer electrolyte, polysiloxane, single‐ion conductor, sodium metal

## Abstract

Sodium‐metal batteries promise higher energy densities than sodium‐ion batteries, but the high reactivity of sodium metal remains a great challenge. Herein, we present a novel polysiloxane‐based single‐ion conducting polymer electrolyte (SIPE). Blended with poly(vinylidene fluoride‐co‐hexafluoropropylene) and infiltrated with small molecular mass organic carbonates, the resulting electrolyte membranes (NaPSiOM) exhibit a high ionic conductivity of 0.2 and 0.4 mS cm^−1^ at 20 and 40°C, respectively, as well as a wide electrochemical stability window (>4.4 V). The NaPSiOM membranes enable an exceptionally stable cycling of symmetric Na║Na cells for over 2000 h and excellent long‐term cycling stability of Na║Na_3_V_2_(PO_4_)_3_ (NVP) cells for over 1000 cycles. Remarkably, these very good performance metrics are well preserved also when increasing the active material mass loading to commercially relevant levels of up to about 18 mg cm^−2^ and when changing the cathode chemistry. In fact, when coupled with a Na_4_Fe_3_(PO_4_)_2_P_2_O_7_ cathode, NaPSiOM enables stable cycling for more than 600 cycles at room temperature in single‐layer pouch cells comprising 23 cm^2^ electrodes, thus, underlining the great potential of NaPSiOM for high‐performance sodium batteries.

## Graphical abstract

We report a polysiloxane‐based single‐ion conducting polymer electrolyte for sodium batteries. This polymer electrolyte exhibits high Na^+^ ion mobility and a wide electrochemical stability window, enabling the long‐term stable cycling of various cathode chemistries – also at room temperature, elevated dis‐/charge rates and active material mass loadings, as well as in pouch cells, thus, underscoring the great potential of this electrolyte.

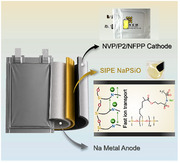

## 1 Introduction

Sodium‐ion batteries (SIBs) have recently attracted significant attention due to the large global abundance of sodium and the potential use of solely sustainable and cost‐effective elements and compounds such as manganese and iron rather than large amounts of nickel and cobalt [1]. Several companies have initiated mass production plans for SIBs, and the first commercial cells have recently been launched [2]. However, the energy density remains well below the currently dominating lithium‐ion technology [3]. One possibility to substantially increase the energy density might be the replacement of the classically used hard carbon (HC) anodes by sodium metal owing to its superior specific capacity of 1165 mAh g^−1^ compared to ca. 300 mAh g^−1^ for the former [4, 5]. Nevertheless, sodium‐metal anodes face critical challenges, including a high reactivity in contact with classically used liquid electrolyte systems such as organic carbonates, resulting in an increasing internal resistance, poor cycling stability, low Coulombic efficiency, and short cycle life of the cells [6, 7, 8]. To address these issues, solid polymer‐based electrolytes have emerged as promising candidates to replace liquid electrolytes [9, 10, 11]. Conventional polymer electrolytes based on, e.g., polyethylene oxide incorporating conducting sodium salts such as NaPF_6_, NaClO_4_, or sodium bis(fluorosulfonyl)imide (NaFSI), typically exhibit low sodium‐ion transference numbers (*t_Na+_*) of less than 0.25 [12, 13, 14], leading to severe concentration gradients at the electrode│electrolyte interfaces, with the risk of dendritic sodium deposition at the negative electrode, poor interfacial stability, and sluggish de‐/sodiation kinetics [15, 16, 17, 18, 19]. As an alternative, single‐ion conducting polymer electrolytes (SIPEs) have been proposed to overcome these challenges by providing cationic transference numbers approaching unity and negligible concentration gradients thanks to the covalent bonding of the anion to the polymer backbone, which leaves the cation as the only mobile species. However, SIPEs typically suffer from limited ionic conductivity at room temperature [19, 20, 21, 22]. This challenge can be overcome by incorporating a certain amount of highly mobile, small molecules with a high dielectric constant such as cyclic carbonates, serving as “molecular transporters” for the alkali‐metal cations [23, 24, 25, 26, 27, 28, 29]. It appears noteworthy that, so far, research on Na^+^ conducting SIPEs remains relatively limited, and only a few examples have been reported recently [30, 31, 32, 33, 34, 35], underlining the need for additional efforts to optimize this promising technology and to promote the development of high‐performance and safe sodium‐metal batteries (SMBs) employing such electrolyte systems. Recently, we reported a polysiloxane‐based SIPE (PSiO) for lithium batteries, demonstrating outstanding performance in combination with nickel‐rich layered oxide‐based cathodes [36]. In this work, we transfer this promising concept to SMBs by adjusting the final ion exchange step for the ionic side chain precursor accordingly. The resulting SIPE, hereinafter referred to as NaPSiOM, exhibits a very high ionic conductivity in combination with a high compatibility with sodium‐metal electrodes, enabling the realization of fast‐charging SMB cells including Na_3_V_2_(PO_4_)_3_ (NVP) positive electrodes with an outstanding capacity retention of 96% after 1000 cycles. This excellent performance is maintained also for high NVP mass loadings of up to 18 mg cm^−2^, while the versatility of this system is further demonstrated by its compatibility with layered sodium transition metal oxides (P2‐type Na_0.67_Mn_0.8_Fe_0.1_Ti_0.1_O_2_, P2‐NMFT) and cost‐effective polyanion cathodes (Na_4_Fe_3_(PO_4_)_2_P_2_O_7_, NFPP) as well as its potential use in SIBs. Moreover, we report the first successful implementation of a Na^+^ conducting SIPE in a large‐format pouch‐cell configuration, delivering stable cycling for over 600 cycles with a capacity retention of >90% at room temperature (RT). This beneficial combination of high‐rate capability, long‐term stability, and scalability highlights the potential of NaPSiOM for high‐performance sodium batteries.

## 2 Results and Discussion

**FIGURE 1:**
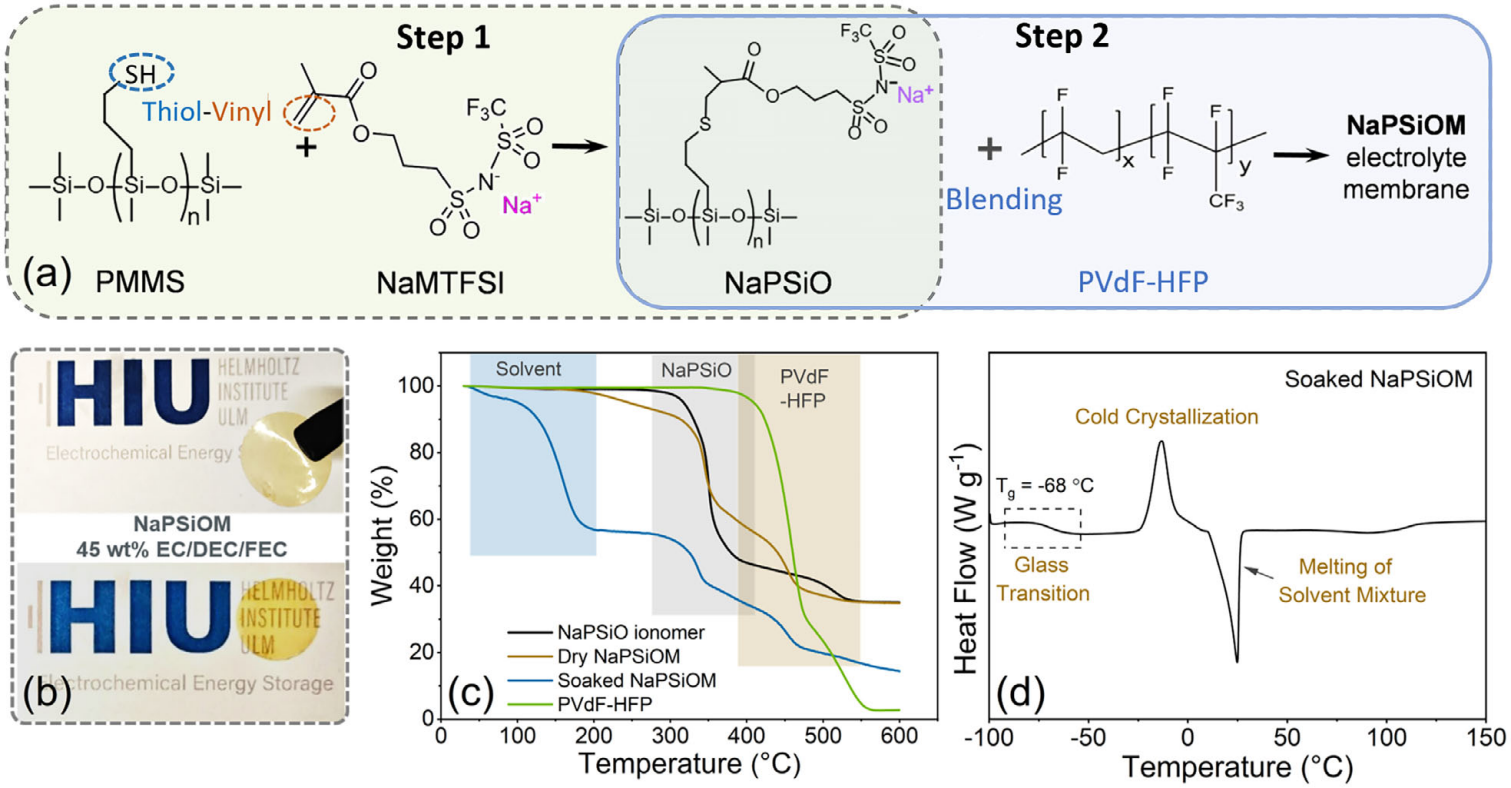
(a) Schematic illustration of the NaPSiO synthesis and the blending with 45 wt.% PVdF‐HFP to achieve the NaPSiOM electrolyte membranes. (b) Photographs of the freestanding NaPSiOM membranes after incorporating the ternary mixture of organic carbonates, i.e., EC/DEC/FEC. (c) TGA data recorded for the NaPSiO ionomer, dry NaPSiOM membranes, the carbonate‐soaked NaPSiOM, and neat PVdF‐HFP serving as reference. (d) DSC analysis of the carbonate‐soaked NaPSiOM recorded within a temperature range from −100°C to 150°C.

The synthesis and chemical characterization of the NaPSiO ionomer and of the corresponding electrolyte membrane (NaPSiOM) is reported in Figure 1. The schematic representation of the synthesis of the NaPSiO ionomer depicted in Figure 1a displays the thiol‐ene “click” reaction between the sodium 1‐[3‐(methacryloyloxy)propylsulfonyl]‐1‐(trifluoromethanesulfonyl)imide (NaMTFSI) side chain and the poly[(mercaptopropyl)methylsiloxane] (PMMS) backbone. The resulting NaPSiO structure relies on the weakly coordinating trifluoromethanesulfonyl anion, which facilitates Na^+^ dissociation and is covalently bonded to the PSiO chain, allowing only the Na^+^ ions to move, while the flexible PMMS backbone benefits from a stable Si─O bond that promotes ion mobility and ensures electrochemical stability toward the positive and negative electrode [37, 38]. The Fourier transform infrared (FT‐IR) analysis depicted in Figure S1a confirms the successful synthesis of the NaMTFSI side‐chain, which presents a spectrum analogous to that previously reported for LiMTFSI [36]. Moreover, the FT‐IR data indicate the complete reaction between NaMTFSI and PMMS, as evidenced by the disappearance of the C═C bond signal at ν = 1640 cm^−1^ in the NaPSiO spectrum from NaMTFSI and the disappearance of the S─H bond at ν = 2560 cm^−1^ from PMMS (see the magnification of the spectra in Figure S1b). This conclusion is further supported by the ^1^H nuclear magnetic resonance (NMR) spectroscopy analyses presented in Figure S2, where the C═C signals at 5.6 and 6.1 ppm from NaMTFSI are not detected for NaPSiO, corroborating the complete monomer conversion. To obtain self‐standing electrolyte membranes, the ionomer was subsequently blended with poly(vinylidene fluoride‐co‐hexafluoropropylene) (PVdF‐HFP; Figure 1a), yielding polymer membranes with a thickness of about 100–150 µm. The analysis via scanning electron microscopy (SEM) in combination with energy‐dispersive X‐ray spectroscopy (EDX), as presented in Figure S3, displays a uniform surface morphology with some pores that presumably benefit the solvent uptake and a homogeneous distribution of the constituent elements (C, F, O, Na, and Si), confirming the intimate mixing of the NaPSiO ionomer with PVdF‐HFP. Subsequently, the mixture of organic carbonates, i.e., ethylene carbonate (EC)/diethylene carbonate (DEC)/fluoroethylene carbonate (FEC) (volume ratio: 48.8:48.8:2.4) was incorporated into the dry NaPSiOM, serving as molecular transporters to facilitate the charge transport. Photographs of these membranes are presented in Figure 1b. Thermogravimetric analysis (TGA) was performed to assess the thermal behavior of the NaPSiO ionomer and of the NaPSiOM membranes, and to determine the solvent uptake of the latter. Figure 1c reports the thermograms obtained, showing for NaPSiO a thermal stability up to about 300°C. PVdF‐HFP is even more stable up to about 400°C, which is in good agreement with previous studies [39, 40]. Interestingly, NaPSiOM exhibits a stepwise mass loss, starting at around 150°C with additional mass loss steps at about 300°C and 400°C, reflecting the thermal decomposition onsets of NaPSiO and PVdF‐HFP. The earlier mass loss observed at 150°C–300°C is attributed to the evaporation of residual dimethyl sulfoxide (DMSO), which was used for the membrane fabrication. In fact, it has been reported earlier already that small traces of high‐boiling‐point solvents such as DMSO, dimethylformamide (DMF), or *N*‐methyl‐2‐pyrrolidone (NMP) commonly used to dissolve PVdF‐HFP, might remain in the sample despite extensive drying [36, 41, 42]. Besides, the TGA of the carbonate‐soaked NaPSiOM allows for the determination of the total content of the carbonate mixture in these electrolyte membranes, i.e., the mass loss prior to the onset for the thermal decomposition, yielding a value of 45 wt.%.

Further characterization of the carbonate‐containing membranes was conducted via differential scanning calorimetry (DSC). The data, presented in Figure 1d, reveal a glass transition temperature (*T_g_*) of −68°C (see also the magnification in Figure S4a), which is substantially lower than the *T_g_* of the dry membranes with 22°C (Figure S4b), indicating that the incorporation of the organic carbonates also has a plasticizing effect on the electrolyte membranes. Additionally, a sharp exothermic peak at around −13°C is observed. To clarify its origin, the DSC data recorded for the dry NaPSiO ionomer, pure EC, and the EC/DEC/FEC solvent mixture were compared in Figure S5. Interestingly, none of the three plots exhibits such a feature, indicating that this peak with an associated enthalpy difference (*ΔH*) of 19.2 J g^−1^ (Equation S3 and Table S1) arises from an interaction between (one of) the solvent(s) and the polymer matrix. In fact, it has been reported earlier the confinement of amorphous EC‐rich domains in a (rigid) polymer network might show such kinetically suppressed, cold crystallization behavior [43]. This assignment is further supported by the subsequent melting peak at about 25°C (*ΔH* = 23.4 J g^−1^, Table S1).

Subsequently, the mechanical properties of the electrolyte membranes were evaluated by measuring the stress–strain curves of the soaked NaPSiOM samples (Figure S6). The corresponding mechanical parameters, i.e., Young's modulus (*E_t_*), the tensile strength (*σ_M_*), and the elongation at break (*ε_M_*), are summarized in Table S2. The soaked NaPSiOM films exhibit a Young's modulus of 0.03–0.06 MPa, a tensile strength of 2.4–3.2 MPa, and an elongation at break of 470%–483%, indicating a highly flexible, yet mechanically robust network, comparable to previously reported values for PVdF‐HFP‐based gel polymer electrolytes [44, 45, 46, 47], but with a markedly higher stretchability, which we may attribute to the presence of the polysiloxane backbone. Such combination of low Young's modulus and high elongation is expected to promote a uniform interfacial contact with the two electrodes, while the high tensile strength enables the preservation of the membrane integrity and contributes to a dendrite‐free sodium deposition at the negative electrode [48].

**FIGURE 2:**
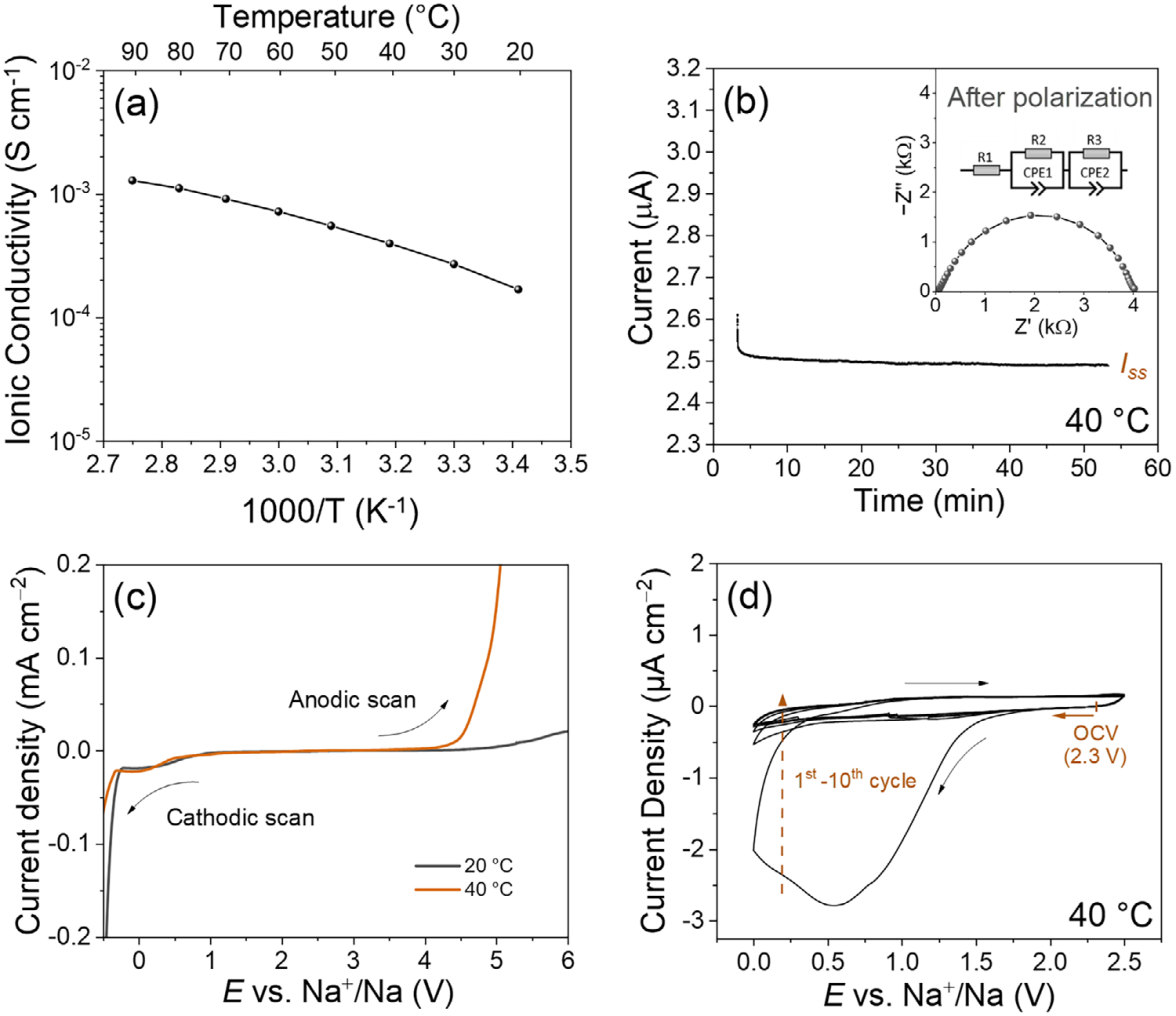
(a) Ionic conductivity as a function of temperature for NaPSiOM. (b) Determination of t_Na+_ using the Watanabe method [51, 53] based on the combination of chronoamperometry (bias voltage: 10 mV) and EIS (frequency range between 1 MHz and 50 mHz, corresponding Nyquist plot reported in inset) performed on a symmetric Na║Na cell at 40°C. (c) Determination of the electrochemical stability window of NaPSiOM by LSV in Na║SS cells at 20°C and 40°C (sweep rate: 1.0 mV s^−1^). (d) CV measurement performed on Na║SS cells between 0 and 2.5 V vs. Na^+^/Na at 40°C (sweep rate: 1.0 mV s^−1^).

The ionic conductivity of NaPSiOM was calculated using Equation S1. The bulk resistance values listed in Table S3 were obtained by non‐linear least squares (NLLS) fitting of the electrochemical impedance spectroscopy (EIS)‐ derived Nyquist plots reported in Figure S7. The Arrhenius‐type plot of the ionic conductivity is displayed in Figure 2a. NaPSiOM shows very high conductivity values of, e.g., 0.2 and 0.4 mS cm^−1^ at 20°C and 40°C, respectively. Generally, the conductivity as a function of temperature shows a typical Vogel–Tammann–Fulcher (VTF) trend (see also the corresponding VTF fitting in Figure S8 and Table S4 obtained by using Equation S4), which is consistent with the analogous Li‐based PSiOM system reported earlier [36], and in line with the expected charge transport from one anionic site to another, aided by the segmental dynamics of the ionomer and the presence of the organic carbonate molecules that are facilitating the dissociation from the anionic groups while serving as additional (highly mobile) coordination sites for the Na^+^ cations. In fact, the FT‐IR spectrum recorded for the soaked NaPSiOM (Figure S9) exhibits the characteristic C═O stretching vibrations at 1803 and 1773 cm^−1^ corresponding to free EC and Na^+^‐coordinated EC molecules, respectively [49, 50]. This strong interaction between the (cyclic) carbonate solvents and the Na^+^ cations corroborates the role of the carbonate molecules as active “molecular transporters,” lowering the energy barrier for ion migration and increasing the ionic conductivity [51].

The nature of the charge transport was further investigated by determining the Na^+^ transference number (*t_Na+_*) by combining chronoamperometry and EIS on symmetric Na║Na cells, as depicted in Figure 2b. We used the method proposed by Watanabe et al. [52] (Equation S2), which differs from the conventional Bruce–Vincent–Evans method [53] by not taking into account the initial current value for the chronoamperometric polarization, whose accurate determination is particularly challenging in the case of highly reactive sodium electrodes, which may lead to unreliable *t_Na+_* values, especially with regard to the very high interfacial resistance observed for this system (see the inset in Figure 2b and the values provided in Table S5) [52, 54]. According to this method, NaPSiOM exhibits a *t_Na+_* of 0.82. While this is a very high value in general, it is significantly lower than the expected *t_Na+_* of unity. Interestingly, this is a common observation for Na^+^ conducting SIPEs, commonly showing a *t_Na+_* of below 0.9 (cf. Table S8). Given that the Li^+^ conducting SIPE reported earlier showed a significantly higher *t_Li+_* of 0.96 (using the Bruce–Vincent–Evans method, though) [36], we may assume that this is linked to the high reactivity of the sodium‐metal electrode, yielding low‐molecular mass decomposition products with a negative charge at the electrode│electrolyte interface. While this certainly deserves an in‐depth investigation – in particular with regard to the fact that this is apparently a common phenomenon – the given value still underlines that charge transport occurs dominantly (if not completely – at least in the bulk) via Na^+^ cations.

To obtain some first insights into the reactivity at the interfaces, we studied the electrochemical stability of the electrolyte by performing linear sweep voltammetry (LSV) on Na║stainless‐steel (SS) cells either from the open circuit voltage (OCV) to 6.0 V vs. Na^+^/Na (anodic scan) or from the OCV to −0.5 V vs. Na^+^/Na (cathodic scan) at 20°C and 40°C. The resulting voltammograms are shown in Figure 2c. When considering a threshold for the evolving current density of 3 µA cm^−2^ as the “stability limit” toward oxidation, the stability was found to be ca. 4.4 V vs. Na^+^/Na at 40°C and up to 4.8 V at 20°C (see Figure S10 for a magnification of relevant area), revealing that any kind of oxidative decomposition is kinetically triggered at elevated temperatures, while the polymer electrolyte membranes generally show a remarkable electrochemical stability toward oxidation.

Toward reduction, the cathodic scan shows a weak current development at potential values below 1.0 V vs Na^+^/Na for both temperatures, indicative of a partial reduction of the electrolyte components (compare the earlier discussion concerning *t_Na+_*), followed by sodium plating below 0 V vs. Na^+^/Na. To further investigate the potential range below 1.0 V, cyclic voltammetry (CV) was performed using the same cell configuration between 0 and 2.5 V vs. Na^+^/Na at 40°C, as displayed in Figure 2d. An irreversible broad signal is observed during the first discharge with an onset potential near 1.5 V vs. Na^+^/Na and centered at about 0.5 V vs. Na^+^/Na (note the different scale of the *y*‐axis in this case), reaching a peak current of −2.8 µA cm^−2^. In contrast, the subsequent CV profiles show an essentially complete overlap with no significant current evolution. The irreversible process observed at low potentials during the first scan is most likely associated with the partial reduction of DMSO traces trapped in the electrolyte membrane, in line with the TGA results in Figure 1c, and an initial reductive decomposition of (some of) the electrolyte components at the interface with the sodium‐metal electrode, while the subsequent observation of essentially no current flow indicates a successful passivation.

**FIGURE 3:**
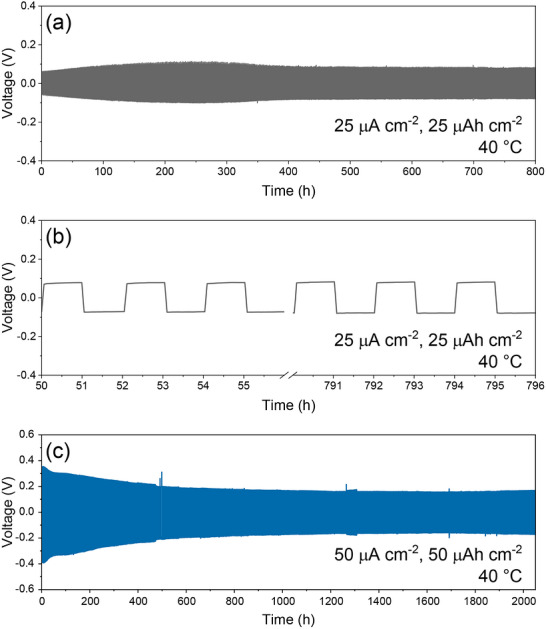
Long‐term galvanostatic plating/stripping experiments performed at 40°C in symmetric Na║Na cells at a current density of (a, b) 25 µA cm^−2^, with a magnification of selected plating/stripping profiles in panel (b), and (c) 50 µA cm^−2^ with a step time of 1 h for each stripping and plating step.

To further investigate the compatibility with sodium‐metal electrodes, the electrolyte membranes were sandwiched between two sodium‐metal electrodes and the resulting symmetric Na║Na cells were subjected to continuous plating/stripping experiments at 40°C. Long‐term measurements were performed using a current densities of 25 µA cm^−2^ (Figure 3a,b) and 50 µA cm^−2^ (Figure 3c), both of which show stable cycling over hundreds and even thousands of hours. In Figure 3a, the initial increase in overpotential is ascribed to the evolution of the SEI layer, becoming more resistive initially, before stabilizing and slightly decreasing again – presumably owing to a slight increase in surface area of the sodium‐metal electrode and/or an enhanced contact with the polymer electrolyte. In fact, when comparing the very early plating/stripping profiles with those recorded after almost 800 h (Figure 3b), one cannot detect any significant difference, highlighting the suitability of this electrolyte system for sodium‐metal electrodes. Besides, the given magnification of the plating/stripping profiles shows a constant voltage response, further corroborating the (essentially) single‐ion conducting behavior and the earlier assumption that any kind of negative charge transport contribution remains limited to the intimate interface between the sodium‐metal electrode and electrolyte [27, 55, 56, 57]. It is noteworthy that the stabilized overpotential is about 80 mV at 25 µA cm^−2^, doubling to ca. 160 mV in the case of 50 µA cm^−2^ (Figure 3c), i.e., twice the current density, thus, following Ohm's law. Additional sodium plating/stripping tests were performed to determine the critical current density (CCD) of the NaPSiOM membrane by stepwise increasing the current density (Figure S11), revealing a CCD of 1.0 mA cm^−2^, which indicates a relatively fast charge transport and transfer.

**FIGURE 4:**
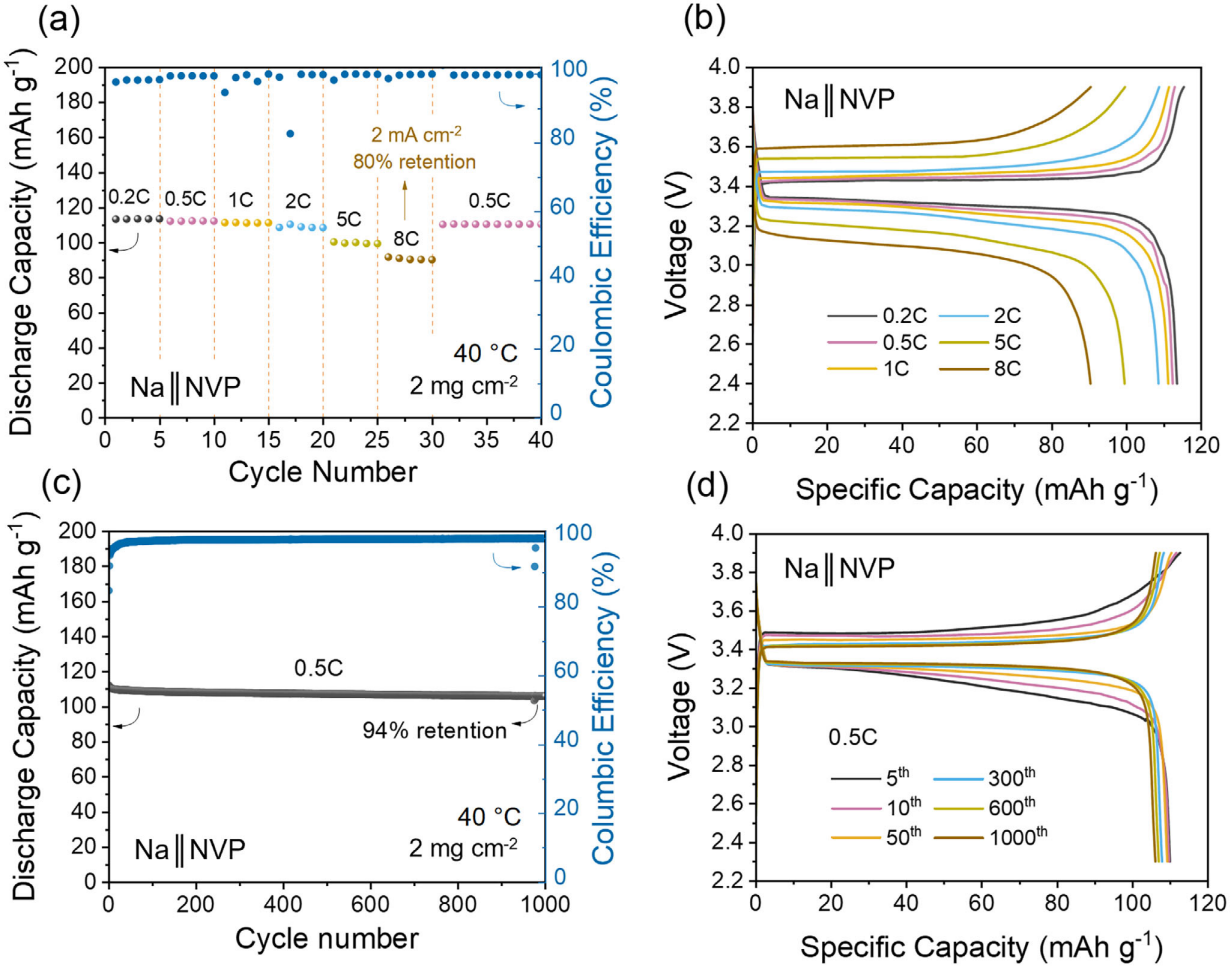
Galvanostatic cycling of Na║NVP cells using NaPSiOM as the electrolyte with NVP electrodes having an active material mass loading of about 2 mg cm^−2^ (T = 40°C; cut‐off voltages: 2.4 and 3.9 V): (a, b) Rate capability test at increasing C rates from 0.2C to 8C, and (c, d) long‐term cycling at a constant C rate of 0.5C (1C = 118 mA g^−1^). The plots of the specific discharge capacity and Coulombic efficiency vs. the cycle number are shown in (a,c), selected dis‐/charge profiles are shown in panel (b,d).

In a next step, we studied the performance in Na║NVP cells to evaluate the applicability of the electrolyte with classically used cell chemistries. The first results in this direction are presented in Figure 4, employing NVP electrodes with an active material mass loading of around 2.0 mg cm^−2^ and setting the temperature to 40°C – somewhat ideal experimental conditions to evaluate the potential performance of the electrolyte as such, without adding other potentially limiting factors at this stage. The investigation of the rate capability of such cells is displayed in Figure 4a,b, increasing the C rate stepwise from 0.2C to 8C. Remarkably, until 2C the decrease in capacity remains negligible. At 0.2C the cell provides a specific capacity of 113 mAh g^−1^, which decreases slightly to 112, 111, and 108 mAh g^−1^ at 0.5C, 1C, and 2C, respectively. At further elevated C rates of 5C and 8C, corresponding to a high current density of 1.2 and 1.9 mA cm^−2^, respectively, though still being well below the limiting current density of 2.7 mA cm^−2^ (Figure S12), the specific capacity decreases to 100 and 91 mAh g^−1^, which is still 88% and 80% of the initial capacity. These results demonstrate that this SIPE is well set also for applications that require (relatively) fast discharging and charging. In fact, the corresponding dis‐/charge profiles (Figure 4b) show a continuous, but rather smooth increase in polarization and the characteristic shape, i.e., the flat potential plateau related to the phase transition and V^3+^/V^4+^ redox couple [58], is well preserved throughout the rate capability test – especially when keeping in mind the pronounced charge transfer resistance at the interface with the sodium‐metal electrode (cf. the inset in Figure 2b and Figure 3).

For the long‐term constant current cycling, we set the C rate to a medium value of 0.5C, and the results are in Figure 4c,d. The capacity remains almost constant for more than 1000 cycles with an initial reversible capacity of 112 and 106 mAh g^−1^ at the end of the test, which corresponds to a capacity retention of 94%. In fact, between the 500th and 1000th cycle, the cell lost only 1 mAh g^−1^, i.e., from 107 to 106 mAh g^−1^, further underlining the excellent performance of this polymer electrolyte. Interestingly, the comparison of selected dis‐/charge profiles upon cycling (Figure 4d and Figure S13) reveals a decreasing overpotential, presumably related to an initial wetting of the NVP electrode with the polymer electrolyte and an increase in surface area of the cycled sodium electrode.

**FIGURE 5:**
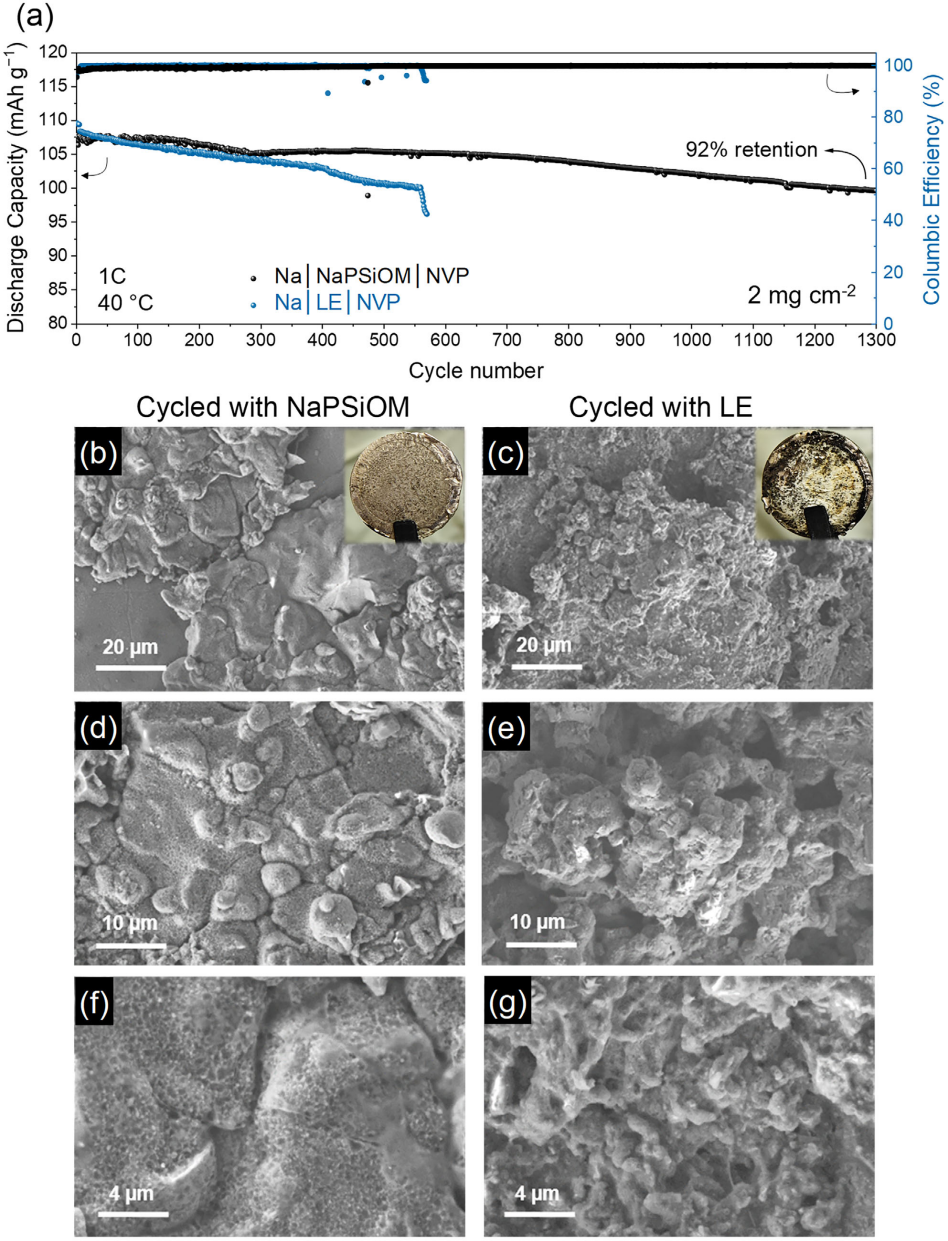
(a) Galvanostatic cycling of Na║NVP cells employing NaPSiOM or a classic organic carbonate‐based liquid electrolyte (LE, 1 m NaPF_6_ in EC/DEC/FEC, 48.8:48.8:2.4 v/v/v) at 1C, with NVP electrodes having an active material mass loading of about 2 mg cm^−2^ (T = 40°C; cut‐off voltages: 2.4 and 3.9 V; 1C = 118 mA g^−1^). (b–g) SEM micrographs acquired for the Na‐metal electrodes after 100 cycles employing (b, d, f) NaPSiOM or (c, e, g) LE as the electrolyte; the insets in panel (b,c) show photographs of the cycled Na electrodes.

To highlight the advantageous impact of the ionomer on this excellent rate capability and cycling stability, we conducted a direct comparison with a liquid electrolyte (LE) comprising the mixture of organic carbonates incorporated also in the polymer membranes (EC/DEC/FEC, 48.8:48.8:2.4, v/v/v) and 1m NaPF_6_, which is an electrolyte composition that is fairly close to classically used organic carbonate‐based liquid electrolytes [59, 60]. Figure 5a shows the discharge capacity vs. cycle number trends of the Na║NVP cells cycled at 1C (T = 40°C), revealing a slightly higher initial capacity for the LE‐based cell (110 mAh g^−1^ compared to 108 mAh g^−1^ in the 1st cycle), most likely due to the higher ionic conductivity of the LE [61]. However, the NaPSiOM‐based cell exhibits a far superior cycling stability, still providing 90% of the initial capacity after 1000 cycles, while the LE‐based cell fades after “only” 570 cycles (still a remarkable value, demonstrating the general suitability of such LE composition in combination with sodium‐metal electrodes). It appears noteworthy that the same trend is observed at a slightly lower C rate of 0.5C (Figure S14), at which the LE‐based cell fades after about 460 cycles, while the NaPSiOM‐based cell shows stable cycling for more than 1000 cycles.

For a fair comparison of the impact of the electrolyte on the sodium‐metal electrode, we cycled new cells employing the two different electrolyte systems for 100 cycles each, before disassembling them in an argon‐filled glove box and transferring the electrodes in an airtight sample holder to the SEM. In fact, already the visual inspection by eye revealed substantial differences. The sodium electrode cycled with NaPSiOM shows a rather uniform surface and the formation of a uniform greyish passivation layer (see the inset in Figure 5b), while the electrodes cycled with the LE exhibit unevenly distributed dark, rather blackish deposits (inset in Figure 5c), indicating substantial electrolyte and electrode degradation and an inhomogeneous sodium plating (and stripping). This first impression is further corroborated by the subsequent ex situ SEM analysis. For NaPSiOM (Figure 5b,d,f), relatively large, micrometer‐sized and bulky sodium deposits are observed, having a rather smooth appearance and covered by a very thin surface layer (Figure 5f). Differently, for LE (Figure 5c,e,g), very porous, high‐surface area surficia structures are observed, even partially dendritic (Figure 5g). The corresponding elemental mapping via EDX is presented in Figures S15 and S16 for the sodium electrodes cycled with LE and NaPSiOM, respectively; along with an overview of the ratio of the different elements in Table S6 (LE) and Table S7 (NaPSiOM). In the case of LE, more than 60% of the detected elemental species, i.e., O, F, C, and P, are indicative of electrolyte decomposition products (Table S6) [62, 63], and Na accounts for only 39% – still being potentially also related to electrolyte decomposition when present as Na_2_CO_3_, NaF, etc., while only three brighter spots in the upper left quarter of the Na map (Figure S15) might refer to metallic sodium. In contrast, the NaPSiOM‐cycled sodium electrode surface exhibits a high Na content of almost 70% and a relatively weak signal for the other elements, indicating a substantially thinner electrolyte‐degradation‐related surface layer. Moreover, it appears interesting to note that the bright spots in the F map are also areas of higher intensity in the O, C, and Si maps, while essentially signal‐free in the Na map (Figure S16), suggesting that there might be some polymer left on the sodium electrode.

**FIGURE 6:**
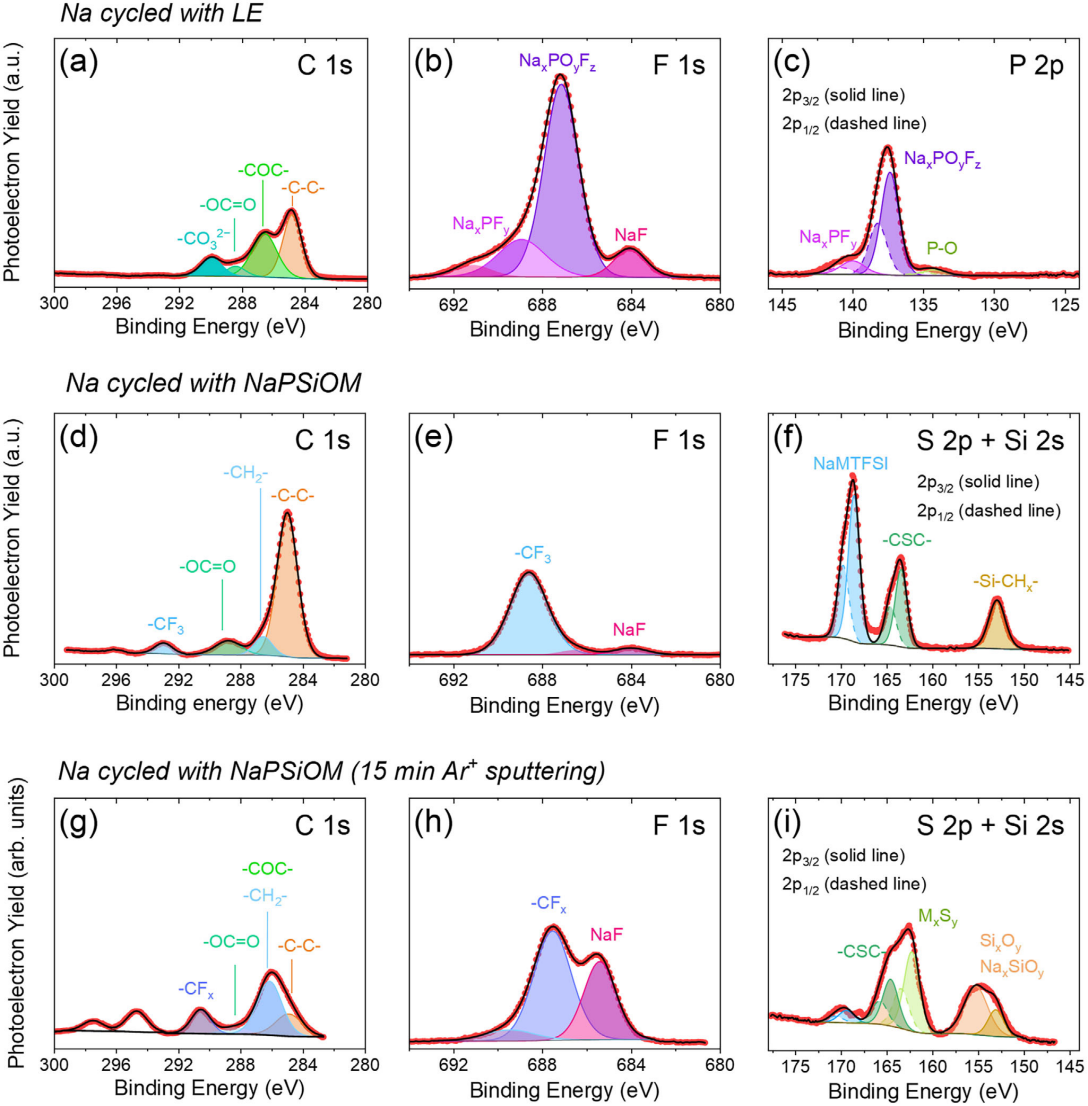
High‐resolution XPS spectra acquired on the surface of the Na‐metal electrodes cycled in Na║NVP cells employing (a–c) LE (C 1s, F 1s, and P 2p) and (d–f) NaPSiOM (C 1s, F 1s, S 2p, and Si 2s) as the electrolyte. (g–i) XPS spectra recorded for the Na‐metal electrodes recovered from the Na║NVP cells employing NaPSiOM as the electrolyte after 15 min of Ar^+^ sputtering (C 1s, F 1s, S 2p, and Si 2s; sputtering rate: 0.8 nm min^−1^). Note that there is a shift in energy for rather insulating compounds such as NaF after Ar^+^ sputtering owing to a significant charge accumulation at the sample surface, which cannot be fully compensated [64, 65, 66, 67].

The formation of any kind of interphase was further investigated via ex situ X‐ray photoelectron spectroscopy (XPS). The C 1s and F 1s detail spectra, as well as the P 2p and S 2p + Si 2s detail spectra for LE and NaPSiOM, respectively, are depicted in Figure 6. The C 1s spectrum of LE (Figure 6a) displays four peaks related to hydrocarbons (C–C, 284.8 eV), ethers (‐COC‐, 286.6 eV), esters (–OC═O, 288.4 eV), and carbonates (–CO_3_^2−^, 290 eV) [68], indicating the reductive decomposition of the electrolyte solvents [69, 70]. Additionally, there is a pronounced decomposition of the conducting salt NaPF_6_ into sodium fluorophosphates (Na_x_PO_y_F_z_) and, to a lesser extent, NaF and Na_x_PF_y_, as evident from the F 1s (Figure 6b) and P 2p (Figure 6c) detail spectra [71]. Differently, the surface of the sodium electrode cycled with NaPSiOM is covered by a chemical species such as hydrocarbons (C–C, 284.8 eV), ‐CH_2_‐ from PVdF‐HFP (286.6 eV), esters (–OC═O, 288.4 eV) from NaPSiO, and highly fluorine‐containing species (‐CF_3_, 293 eV) from PVdF‐HFP and/or the TFSI‐type anion (Figure 6d,e) [68], suggesting that some residues of the polymer electrolyte membrane remained on the sodium electrode, indicating a very strong contact. Also the S 2p and Si 2s spectral features appear to confirm the presence of NaPSiOM membrane residues, exhibiting peaks related to the TFSI anion as well as ‐CSC‐ and ‐Si‐CH_x_‐ species from the NaPSiO polymer backbone (Figure 6f) [72]. Nevertheless, the F 1s region (Figure 6e) also indicates the reduction of the TFSI anion, as suggested by a small concentration of NaF. Therefore, to further analyze the SEI formed on the sodium electrode, 15 min of Ar^+^ sputtering was conducted to remove the external NaPSiOM layer and study the intimate interphase between the sodium electrode and the NaPSiOM electrolyte membrane. The C 1s spectrum (Figure 6g) indicates the removal of any residual NaPSiOM and reveals the formation of fluorinated species due to the decomposition of PVdF‐HPF and/or the TFSI anionic group, as well as the presence of ether and ester groups from the reduction of the organic carbonates. Meanwhile, the F 1s spectrum (Figure 6h) shows a higher concentration of NaF in the inner SEI layer, accompanied by other, potentially beneficial inorganic species such as Na_x_SiO_y_ and Na_2_S identified in the S 2p and/or Si 2s spectrum (Figure 6i). These results indicate that sodium metal cycled with NaPSiOM forms a more inorganic‐rich SEI. The presence of Na_2_S is expected to inhibit Na_2_CO_3_ formation [73], while Na_x_SiO_y_ can enhance the SEI mechanical robustness [74, 75]. Together, these inorganic components, particularly Na_x_SiO_y_ and NaF, promote a fast and homogeneous Na^+^ transport across the interface and interphase, which is consistent with the stable Na plating/stripping behavior (Figure 3) and long‐term stable cycling performance in Na║NVP cells (Figures 4 and 5).

**FIGURE 7:**
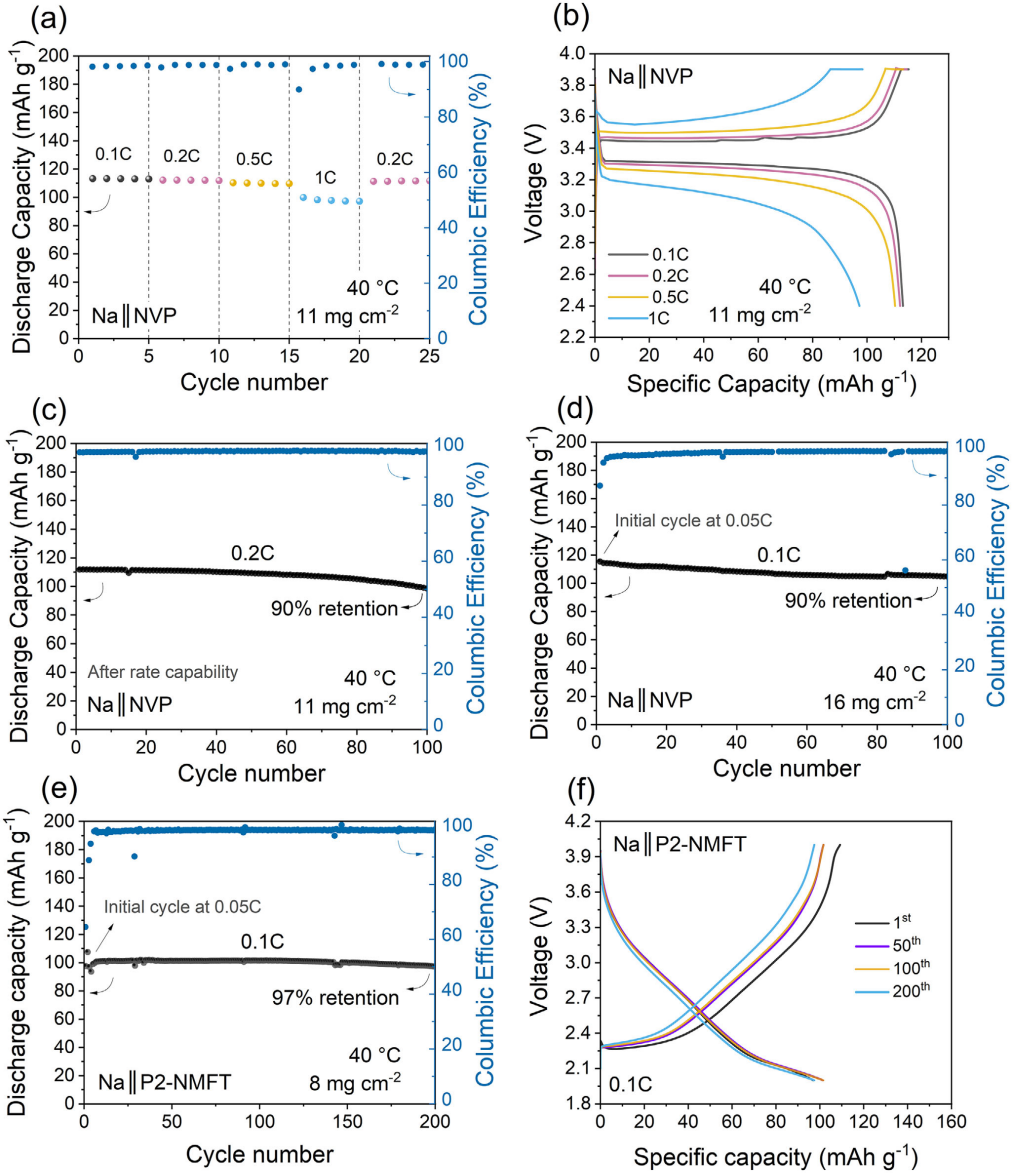
(a–c) Galvanostatic cycling of Na│NaPSiOM│NVP cells with an active material mass loading of ca. 11 mg cm^−2^ (T = 40°C; cut‐off voltages: 2.4 and 3.9 V; 1C = 118 mA g^−1^): (a, b) rate capability test with (a) the plot of the specific discharge capacity and Coulombic efficiency vs. the cycle number and (b) selected dis‐/charge profiles; (c) constant current cycling at 0.2C after the rate capability test shown in panels (a, b) (selected dis‐/charge profiles are presented in Figure S17). (d) Galvanostatic cycling of Na│NaPSiOM│NVP cells with an active material mass loading of ca. 16 mg cm^−2^ (selected dis‐/charge profiles are presented in Figure S18). (e, f) Galvanostatic cycling of Na│NaPSiOM│P2‐NMFT cells employing P2‐NMFT electrodes with an active material mass loading of about 8 mg cm^−2^ cycled at 0.1C (1C = 263 mA g^−1^; T = 40°C; cut‐off voltages: 2.0 and 4.0 V), with (e) the plot of the specific discharge capacity and Coulombic efficiency vs. the cycle number and (f) selected dis‐/charge profiles.

The potential practical applicability of NaPSiOM was further evaluated by galvanostatic cycling of Na║NVP cells applying more challenging experimental conditions such as elevated cathode mass loadings. Figure 7a shows the rate capability test performed on Na║NVP cells containing NVP electrodes with an active material mass loading of around 11 mg cm^−2^, exhibiting an impressive capacity retention with reversible capacities of 113, 111, and 110 mAh g^−1^ at 0.1C, 0.2C, and 0.5C, respectively, which are comparable to those achieved for the low‐mass loading NVP cathodes. Even when increasing the C rate to 1C, the cell exhibits a capacity above 100 mAh g^−1^, reflecting the remarkable Na^+^ transport kinetics of the NaPSiOM membrane. The corresponding dis‐/charge profiles, depicted in Figure 7b, show minimal polarization until 1C, for which a short overpotential peak is observed at the beginning of the charge step, which can be attributed to ion‐transport limitations in the system, e.g., owing to a still optimizable ion conduction in the NVP electrode, and/or a higher nucleation overpotential for the sodium plating on the negative electrode at such elevated current densities of 1.3 mA cm^−2^. The constant current cycling at 0.2C after the rate capability test is shown in Figure 7c, displaying an initial capacity of 112 mAh g^−1^, in line with the value delivered during the rate capability test, and a capacity retention of 90% after 100 cycles. The minor fading that is observed after about 80 cycles comes along with an increasing overpotential, as apparent from the dis‐/charge profiles presented in Figure S17, which indicates that the failure might be related to a loss of interfacial contact – especially when considering the excellent performance and long‐term cycling stability of the low‐mass loading electrodes for more than 1000 cycles (Figure 4c,d). In fact, when further increasing the active material mass loading of the NVP electrodes to around 16 mg cm^−2^, while decreasing the C rate to 0.1C, leads to an even better cycling stability with a capacity retention of 91% after 100 cycles and an initial specific capacity of 114 mAh g^−^^1^ (Figure 7d), and the corresponding dis‐/charge profiles do not show any significant increase in polarization (Figure S18). When further increasing the NVP active material mass loading to ca. 18 mg cm^−2^ (Figure S19), the cells provide a decent performance at 0.05C (113 mAh g^−1^), 0.1C (112 mAh g^−1^), and 0.2C (111 mAh g^−1^), as apparent from the initial rate capability test, while the capacity starts fading when increasing the C rate to 0.5C, and at 1C the specific capacity drops to less than 40 mAh g^−1^. However, it well recovers to the initial capacity when decreasing the C rate back to 0.1C for the subsequent constant current cycling, providing a capacity retention of 90% after 90 cycles. In sum, these findings indicate that the major limitation at such high mass loadings are kinetic factors related to the electrode design and the charge transport within the electrode, which will certainly benefit from further optimization and advanced electrode engineering. Finally, we also tested the impact of a lower operating temperature of 20°C. The cells show a very stable cycling for more than 350 cycles at 0.5C, with a capacity retention of 98% of the initial 102 mAh g^−1^ and a remarkable average Coulombic efficiency of more than 99% (Figure S20).

Besides, we extended our investigation also to layered sodium transition metal oxides as active material for the positive electrode, using exemplarily P2‐type Na_0.67_Mn_0.8_Fe_0.1_Ti_0.1_O_2_ (P2‐NMFT) owing to its good performance in combination with conventional organic carbonate‐based and ionic liquid‐based electrolytes [76]. Figure 7e shows the capacity delivered by the Na│NaPSiOM│P2‐NMFT cell as function of the cycle number at a constant C rate of 0.1C (1C = 263 mA g^−1^; T = 40°C). The P2‐NMFT electrodes had an active material loading of about 8 mg cm^−2^. Initially, the capacity increases slightly over the first 6 cycles to reach a value of 101 mAh g^−1^ at the 7th cycle. The capacity retention after the 200th cycles is as high as 97%, a value that is at least among the best reported so far for layered oxides in combination with polymer‐based electrolytes [77, 78, 79], and superior to the results previously reported for this material [80]. The absolute specific capacity, though, is lower than what has been reported for the liquid electrolyte systems with more than 140 mAh g^−1^ [80], which is likely originating from the higher ionic conductivity, the higher electrode active material mass loading used herein (8 vs. 2 mg cm^−2^), and the inferior electrode wetting by the polymer electrolyte compared to the liquid systems, which can easily penetrate the electrode porosity, thus, facilitating the charge transport within the electrode. In fact, the comparison of the corresponding dis‐/charge profiles (Figure 7f) with those reported earlier [80], reveals a 0.2‐V‐greater polarization for the data reported herein, underlining the facilitated charge transfer kinetics for liquid electrolyte systems and, once more, the need to advance the electrode design and architecture when using polymer‐based electrolyte systems.

**FIGURE 8:**
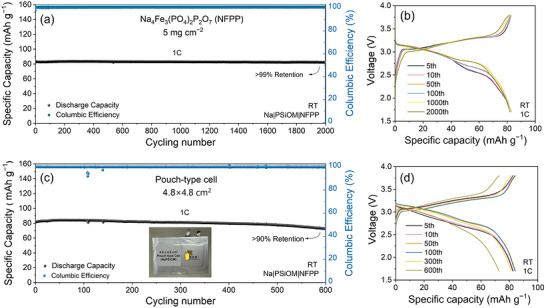
Galvanostatic cycling of Na│NaPSiOM│NFPP cells employing NFPP electrodes with an active material mass loading of about 5 mg cm^−2^ cycled at 1C (1C = 129 mA g^−1^) and 25°C (RT) with cut‐off voltages of 1.7 and 3.8 V in (a, b) coin cells and (c, d) large‐format pouch‐cells, with a plot of (a, c) the specific discharge capacity and Coulombic efficiency vs. the cycle number and (b, d) selected dis‐/charge profiles (the inset in (c) shows a photograph of the large‐format pouch cell).

To further assess the potential use of NaPSiOM, we investigated cells employing a polyanion‐structured Na_4_Fe_3_(PO_4_)_2_P_2_O_7_ (NFPP) cathode at room temperature (RT) in both coin cell and single‐layer pouch cell configuration (Figure 8). The Na│NaPSiOM│NFPP coin cells demonstrate an outstanding cycling stability with a capacity retention of >99% after more than 2,000 cycles at 1C (1C = 129 mA g^−1^; Figure 8a), which represents one of the most stable cycling performances reported so far for SMBs employing SIPEs. The specific capacity is somewhat lower with ca. 83 mAh g^−1^ compared to previous studies [81, 82], which is presumably due to the slower ion‐transport kinetics at RT in NaPSiOM compared to classic liquid electrolytes. The corresponding dis‐/charge profiles reveal only very minor changes from the 5th to the 2000th cycle (Figure 8b), highlighting the excellent stability of the electrolyte in such cell chemistry.

Remarkably, also the large‐format pouch cells comprising NFPP electrodes with a size of 4.8 × 4.8 cm^2^ demonstrate an excellent cycling stability with a capacity retention of >90% after 600 cycles at 1C and at RT (Figure 8c). The corresponding dis‐/charge profiles indicate a slight increase in overpotential and decrease in capacity over the course of the 600 cycles (Figure 8d), suggesting that the cell assembly, electronic contacting, and the long‐term interfacial contact might be further improved for this larger cell format. We may note, though, that this represents the first successful implementation of an Na^+^ conducting SIPE in a pouch‐cell configuration, to the best of our knowledge.

An overview of and comparison with the current state of the art is provided in Table S8. Generally, our results are highly competitive, if not superior to the state of the art. Moreover, our work first reports promising cycling performance also for commercially relevant active material mass loadings of up to about 18 mg cm^−2^ – besides the first report of such large‐format pouch cells. The superior cycling stability observed for cells cycled at both RT and 40°C further highlights the operational flexibility of NaPSiOM for practical applications. This is particularly noteworthy for Na‐metal batteries, which are generally more challenging to stabilize at elevated temperatures due to the higher reactivity of Na metal with the electrolyte and other cell components [83].

**FIGURE 9:**
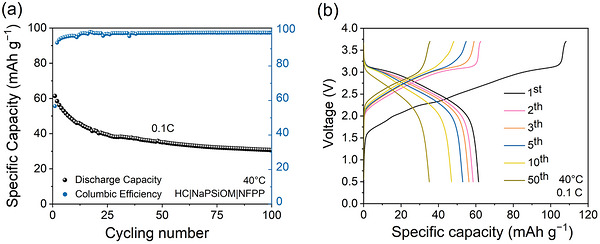
Galvanostatic cycling of HC│NaPSiOM│NFPP sodium‐ion cells employing an NFPP cathode and a hard carbon (HC) anode with an N/P ratio of 1.1, cycled at 0.1C (1C = 129 mA g^−1^) and 40°C (cut‐off voltages: 0.5 and 3.7 V): (a) Plot of the specific discharge capacity and Coulombic efficiency vs. the cycle number and (b) selected dis‐/charge profiles.

Finally, we evaluated the performance of NaPSiOM also in proof‐of‐concept sodium‐ion cells employing an HC anode (i.e., HC│NaPSiOM│NFPP) to investigate the potential use of this new electrolyte system also beyond the combination with Na‐metal electrodes (Figure 9) [84]. We may note that this is more challenging, in fact, having two porous electrodes, especially at the negative electrode where even traces of gaseous decomposition products result in a significant contacting issue. These very preliminary tests (and, to the best of our knowledge, also first attempts in this direction) reveal an initial Coulombic efficiency of 70%, which is somewhat lower than the values reported for classic liquid electrolytes [85], while the shape of the charge profile of the HC anode generally reveals the commonly expected features (Figure S21). The capacity of the sodium‐ion cell decreases rather rapidly at the beginning before eventually stabilizing after about 25 cycles along with the Coulombic efficiency eventually of around 99.3% (Figure 9a), which appears to be linked to an increase in polarization and contact loss (Figure 9b). As stated earlier, achieving a long‐term intimate contact is particularly challenging when incorporating a polymer‐based electrolyte into cells with two porous electrodes. Nevertheless, these findings indicate that NaPSiOM might be also considered as replacement for liquid electrolytes in sodium‐ion cells following further improvement.

## 3 Conclusion

A novel polysiloxane‐based sodium single‐ion conducting polymer electrolyte (NaPSiOM) has been successfully synthesized, providing a high ionic conductivity of 0.4 mS cm^−1^ and a wide electrochemical stability window up to 4.4 V at 40°C. Symmetric Na║Na cells incorporating this new polymer electrolyte show exceptional cycling stability for more than 2000 h. Furthermore, Na│NaPSiOM│NVP cells display outstanding rate capability up to 8C (∼1.9 mA cm^−2^), exceptional cycle life with up to more than 1000 cycles with negligible capacity decay, and very good performance also when elevating the active material mass loading to about 18 mg cm^−2^ or lowering the operating temperature to 20°C with 98% capacity retention after 350 cycles. The great potential of NaPSiOM is further demonstrated by the successful application with layered P2‐type NMFT and cost‐effective NFPP cathodes, delivering exceptional long‐term cycling stability also at ambient temperatures. Notably, the implementation in long‐term stable large‐format Na║NFPP pouch cells and HC║NFPP sodium‐ion cells further highlights the potential of this Na^+^ conducting SIPE as a highly promising electrolyte candidate for safe, durable, and high‐performance sodium batteries.

## Conflicts of Interest

The authors declare no conflicts of interest.

## Supporting information




**Supporting File**: advs76457‐sup‐0001‐SuppMat.docx.

## Data Availability

The data that support the findings of this study are available from the corresponding author upon reasonable request.
