# Supplementary material for: Polysiloxane‐Based Single‐Ion Conducting Polymer Electrolyte for Sodium Batteries
Source: Adv Sci (Weinh). 2026 Jul 14:e00077. Online ahead of print. doi: 10.1002/advs.202600077 (PMC13367105; doi:10.1002/advs.202600077)
Supplement: Supplementary file 1 — Supporting File: advs76457‐sup‐0001‐SuppMat.docx. [file ADVS-9999-e00077-s001.docx]

Supporting Information

**Polysiloxane-Based Single-Ion Conducting Polymer Electrolyte**

**for Sodium Batteries**

Yixuan Guo^1,2,‡^, Hyokyeong Kang^3,‡^, Maider Zarrabeitia^1,2^, Ludovica Roselli^1.2^, Hyerim Kim^3^, Vittorio Marangon^1,2,^*, Jang-Yeon Hwang^3,4,^*, and Dominic Bresser^1,2.5,^*

*^1^ Helmholtz Institute Ulm (HIU), Helmholtzstrasse 11, 89081 Ulm, Germany*

*^2^ Karlsruhe Institute of Technology (KIT), P.O. Box 3640, 76021 Karlsruhe, Germany*

*^3^* *Department of Energy Engineering, Hanyang University, Seoul 04763, Republic of Korea*

*^4^ Department of Battery Engineering, Hanyang University, Seoul 04763, Republic of Korea*

*^5^ Ulm University (UUlm), 89069 Ulm, Germany*

***Corresponding authors:**

[dominic.bresser@kit.edu](mailto:dominic.bresser@kit.edu); [jangyeonhw@hanyang.ac.kr](mailto:jangyeonhw@hanyang.ac.kr); [vittorio.marangon@kit.edu](mailto:vittorio.marangon@kit.edu)

‡ These authors contributed equally.

**Keywords:** single-ion conductor; polymer electrolyte; polysiloxane; sodium metal; battery

**Experimental Section**

*1 NaPSiO Synthesis and Basic Characterization*

The synthesis of sodium (3-methacryloyloxypropylsulfonyl)(trifluoromethylsulfonyl)imide (NaMTFSI) was carried out following a procedure analogous to that previously reported for LiMTFSI,^[1–3]^ simply using sodium hydride (NaH) as the ion-exchange agent in the final step. NaMTFSI was subsequently used to synthesize the NaPSiO ionomer via a thiol–ene click reaction. In detail, 1.76 g NaMTFSI (4.88 mmol) and 0.66 g poly[(mercaptopropyl)methylsiloxane] (PMMS, SMS-992, Mw = 4,000–7,000, 75–150 cSt; Gelest) were dissolved in 10 mL anhydrous tetrahydrofuran (THF). The resulting solution was stirred under argon atmosphere at 65 °C for 14 h. Subsequently, the solvent was removed via rotary evaporation under reduced pressure (<40 mbar) and the product was reprecipitated in an acetone/dichloromethane solution (v/v = 1:9). The final product was collected as a pale yellow solid and dried under vacuum at 80 °C for 24 h to achieve the purified NaPSiO-based ionomer (yield ~64%). The composition and the successful synthesis of final product was confirmed by Fourier transform infrared (FT-IR) spectroscopy using a Spectrum Two spectrometer (Perkin-Elmer) and ^1^H nuclear magnetic resonance (NMR) spectroscopy performed on a Bruker DMX400 NMR spectrometer.

*2 Electrolyte Membrane Preparation*

The NaPSiO-based electrolyte membrane (NaPSiOM) was prepared by blending the NaPSiO ionomer with poly(vinylidene fluoride-co-hexafluoropropylene) (PVdF-HFP, Sigma-Aldrich) at a weight ratio of 55:45 using the solvent casting method. Dimethyl sulfoxide (DMSO) was used to dissolve the polymers achieving a transparent solution, which was subsequently cast into a Teflon Petri dish (diameter of 6.5 cm) and dried at 80 °C to obtain a uniform membrane. The resulting dry membrane was punched into 14 mm-diameter discs and further dried under high vacuum (<10^−2^ bar) for 48 h to remove any residual solvent. Prior to cell assembly, the dry membranes were swollen in the glovebox with a ternary organic carbonate mixture composed of ethylene carbonate (EC), diethyl carbonate (DEC), and fluoroethylene carbonate (FEC) in a volume ratio of 48.8:48.8:2.4, yielding flexible, free-standing NaPSiOM electrolyte membranes. The preparation of large NaPSiOM membranes (4.8×4.8 cm^2^) followed the same procedure as used for the smaller polymer disks. The quantification of the solvent content and the investigation of the thermal behavior of the electrolyte membranes were conducted via thermogravimetric analysis (TGA) using a Netzsch TG 209 F1 instrument under a N_2_/O_2_ (v/v = 1:1) flow between 25 and 600 °C, applying a heating rate of 5 °C min^−1^. Differential scanning calorimetry (DSC) was conducted for the determination of the *T_g_* on a TA Instruments Q2000 analyzer from -100 to 150 °C, where the second heating cycle was used for the determination of the *T_g_*. Scan rates of either 5 and 10 °C min^−1^ were used for the dry and carbonates-infiltrated electrolyte membranes samples, respectively. The mechanical properties of the soaked NaPSiOM membranes were characterized using a universal testing machine (ZwickiLine, ZwickRoell, Germany), equipped with a high-precision load cell and operated via the TestXpert III software. According to the ISO 527-3 standard, the samples were prepared in a dumbbell shape with a total length of 40 mm, a gauge length of 20 mm, and a width of approximately 3–4 mm. The thickness of each film (typically 100–120 µm) was measured using a digital micrometer for accurate stress calculation. Tensile tests were conducted at a pre-test speed of 1 mm min^-1^ for the modulus determination, followed by a constant extension rate of 10 mm min^-1^ until fracturing. At least three specimens were tested for each sample to ensure reproducibility.

*3 Electrode Preparation*

Na_3_V_2_(PO_4_)_3_ (NVP)-based electrodes with mass loading of approximately 2 mg cm^−2^ were prepared by dispersing the NVP powder (MTI corporation, 20 µm), carbon black (C65, Timcal), and poly(vinylidene fluoride) (PVdF, Solvay) in *N*-methyl-2-pyrrolidone (NMP, Sigma-Aldrich) in a weight ratio of 80:10:10. The mixture was stirred overnight at room temperature to obtain a homogeneous slurry, which was then cast onto aluminum foil using a lab-scale doctor blade with a 120 µm blade-height, and subsequently dried at 80 °C overnight. The resulting electrode tape was punched into 12 mm-diameter discs, which were pressed at 5 t cm^−2^ for 10 s and finally dried under vacuum (<10^−2^ bar) for 12 h before being transferred in an Ar-filled glovebox (MBraun, H_2_O and O_2_ content < 0.1 ppm). For the high-mass-loading cathodes, the NaPSiO ionomer was added to the electrode slurry to ensure a suitable ionic conductivity within the electrode. The weight ratio of the electrode components NVP, C65, PVdF, and NaPSiO was 75:10:8:7. A blade-height of 500–800 µm was used for the slurry casting, yielding active material mass loadings in the range from 10 to 18 mg cm^−2^.

P2-type Na_0.67_Mn_0.8_Fe_0.1_Ti_0.1_O_2_ (P2-NMFT) was synthesized via a solid-state method, using stochiometric amounts of monohydrate Na_2_CO_3_ (≥99.5%, Sigma-Aldrich), Mn_2_O_3_ (98%, Thermo Scientific), Fe_2_O_3_ (≥99%, Sigma-Aldrich), and TiO_2_ (99.6%, Thermo Scientific). The obtained powder was annealed at 1,000 °C for 6 h under ambient conditions, then cooled to 80 °C and transferred to a dry room (dew point ca. -80 °C) to prevent any contact with the atmosphere. The P2-NMFT electrodes were prepared by mixing 80 wt.% of active material with 10 wt.% Super C65 (Timcal), and 10% PVdF in 55 wt.% NMP. The slurry was cast on battery-grade Al foil and dried in an oven at 80 °C. 12 mm-diameter electrodes were punched and pressed at 5 t cm^−2^ for 30 s, and subsequently dried under vacuum at 120 °C. The final P2-NMFT active material loading of the electrodes was around 8 mg cm^−2^.

Na_4_Fe_3_(PO_4_)_2_P_2_O_7_ (NFPP) was synthesized via a spray-drying method. Stoichiometric amounts of Fe(NO_3_)_3_·9H_2_O (≥99%, Sigma-Aldrich) and NaH_2_PO_4_ (≥99.5%, Sigma-Aldrich) as well as 0.014 mol of citric acid (≥98%, Sigma-Aldrich) were dissolved in 30 mL deionized water and stirred for 1 h using a magnetic stirrer to obtain a homogeneous precursor solution. The resulting solution was then spray-dried to yield the precursor. The precursor was subsequently calcined in a tubular furnace under an Ar/H_2_ (H_2_ 3.9 wt.%) atmosphere. The heat treatment was carried out in two-steps: for the first step, the powders were heated to 300 °C at a rate of 2 °C min^-1^ and the maximum temperature was kept constant for 3 h; for the second step, the material was further heated to 550 °C at the same heating rate and calcined for 12 h. The corresponding electrodes were prepared by blending the active material (80 wt.%), Super P (Timcal, 5 wt.%) and KS6 (Timcal, 5 wt.%) conductive carbons, as well as PVdF (10 wt.%) in NMP to form a uniform slurry. The slurry was cast onto C-coated Al foil, followed by vacuum drying at 110 °C for 12 h to remove any residual solvent and moisture. The dried electrodes were roll-pressed at 80 °C and cut in 12 mm-diameter discs and 4.8×4.8 cm^2^ tapes, having an average active material mass loading of about 5 mg cm^-2^.

Sodium-metal anodes were prepared in an Ar-filled glovebox by rolling and punching sodium rods (Acros, 99.5%) into discs with a diameter of 14 mm. The thickness of the sodium-metal discs was controlled to be around 500 µm.

The hard carbon (HC) electrodes were prepared by mixing 87 wt.% HC (AHC-3, AEKYUNG CHEMICAL CO.), 3 wt.% Super P conductive carbon, and 10 wt.% PVdF in NMP. The resulting slurry was cast onto carbon-coated copper foil and dried at 110 °C for 12 h in a vacuum oven. The electrodes were roll–pressed and then punched in a glovebox to obtain 12 mm-diameter discs with an average active material mass loading of around 1 mg cm^-2^.

*4 Electrochemical Characterization*

All electrochemical measurements were performed in CR2032 coin-type cells (MTI), which were assembled in an Ar-filled glovebox. The ionic conductivity (*σ*) of the NaPSiOM electrolyte was determined via electrochemical impedance spectroscopy (EIS) performed on symmetric stainless-steel (SS)║SS cells between 1 MHz and 0.1 Hz using a Solartron Impedance Analyzer (ModuLab XM MTS, Ametek Scientific Instruments). The temperature was controlled between 20 and 80 °C with a climatic chamber, and the ionic conductivity for each temperature was calculated using the following equation:

$\sigma=\frac{d}{RS}$ (S1)

where d and S are the thickness and the geometric area of the electrolyte membrane, respectively, and 𝑅 is the bulk resistance obtained from the high-frequency intercept of the Nyquist plot.

The sodium-ion transference number (*t_Na+_*) of the electrolyte was determined via the Watanabe method using a combination of EIS (1 Mhz – 50 mHz frequency range) and chronoamperometry (10 mV for 50 min) performed on symmetric Na║Na cells at 40 °C employing a BioLogic VMP3e instrument.^[4]^ Accordingly, *t_Na+_* was calculated using the following equation:

$t_{{Na}^{+}}=\frac{I_{ss}R_{b}}{\Delta V-I_{ss}R_{i}}$ (S2)

where *ΔV* is the applied voltage, *I_ss_* is the steady-state current measured at the end of the chronoamperometry test, while *R_i_* and *R_b_* are the steady-state interfacial resistance and the bulk resistance of the electrolyte, respectively, obtained by the Nyquist plot of the EIS data recorded after the chronoamperometric measurement.

The electrochemical stability window of NaPSiOM was investigated at 20 °C and 40 °C using Na║SS cells by performing linear sweep voltammetry (LSV) measurements either from open circuit voltage (OCV) to -0.5 V vs. Na^+^/Na (cathodic scan) or from the OCV to 6.0 V vs. Na^+^/Na (anodic scan) using a sweep rate of 0.1 mV s^−1^. Cyclic voltammetry (CV) was conducted at 40 °C for 10 cycles at 1.0 mV s^−1^ from OCV to 0 V vs. Na^+^/Na for the first cyclic sweep and between 0 and 2.5 V vs. Na^+^/Na for the subsequent cyclic sweeps, using the same cell configuration. The limiting current density (LCD) of the NaPSiOM electrolyte was determined at 40 °C by applying a voltage scan with a sweep rate of 0.02 mV s^−1^ using symmetric Na║Na cells. Sodium plating/stripping tests were carried out at 40 °C via on symmetric Na║Na cells, applying either an increasing current density from 0.025 to 1.0 mA cm^−2^ to determine the critical current density (CCD) or a constant current density of 0.05 mA cm^−2^ or 0.025 mA cm^−2^, setting the step time to 1 h for each plating and stripping step. All the voltammetry and EIS measurements were conducted using a BioLogic VMP3e instrument.

The resistance values obtained by EIS were calculated via non-linear least squares (NLLS) fitting of the Nyquist plots using the RelaxIS software (rhd instruments). The equivalent circuit model for the NLLS fitting consisted of a bulk resistance (*R_b_*), indicated by the high-frequency intercept of the spectra with the real-impedance axis (*x*-axis), *R_n_Q_n_* elements for the high/medium frequency semicircles, and an *R_w_Q_w_* element related to the finite-length Warburg-type Na^+^ diffusion represented by the low-frequency semicircle; note that the number and type of circuit elements depend on the eventual shape of the Nyquist plot. The interfacial resistance *R_i_* was obtained by the sum of the *R_n_* values, which were determined by the amplitude of each high/medium frequency semicircles identified through NLLS fitting. Only fittings with a χ^2^ value lower than 10^−3^ were accepted.

Galvanostatic cycling of Na║NVP cells was carried out in the 2.4–3.9 V voltage range. Na│NaPSiOM│NVP cells using NVP electrodes with an active material mass loading of about 2 mg cm^−2^ were subjected to increasing C rates from 0.2C to 8C, varying the C rate every 5 cycles and eventually decreasing it back to 0.5C after 30 cycles, or to a constant C rate of 0.5C or 1C (1C = 118 mA g^−1^). The measurements were performed at 40 °C, if not stated differently. For the measurements at 20 °C, the active material mass loading was about 1 mg cm^−2^. Electrodes with elevated mass loadings of ca. 11 mg cm^−2^ were employed in Na│NaPSiOM│NVP cells, which were subjected to galvanostatic cycling at 40 °C at increasing C rates between 0.1C and 1C by varying the current every 5 cycles, and decreasing it back to 0.2C after 20 cycles for additional long-term cycling at the same C rate. Long-term galvanostatic cycling was performed at 40 °C using a NVP mass loadings of ca. 16 mg cm^−2^ with a constant C rate of 0.1C, applied after one activation cycle at 0.05C. For NVP electrodes with a very high active material mass loading of ca. 18 mg cm^−2^, a rate capability test was performed at 40 °C, applying C rates from 0.05C up to 1C before decreasing the C rate back to 0.1C after 22 cycles for additional long-term cycling. The cells using an NVP mass loading >10 mg cm^−2^ were cycled by adding a constant-current/constant-voltage step at the end of the charge step at 3.9 V for 5 h. The cells using NVP mass loadings of 16 and 18 mg cm^−2^ benefitted from the addition of 10 µL of the EC/DEC/FEC solution directly cast on the NVP cathode during assembly to ensure a proper wetting of the electrodes. For a direct comparison with a liquid electrolyte (LE), Na│LE│NVP cells with an active material mass loading of 2 mg cm^−2^ were galvanostatically cycled at a constant C rate of either 0.5C or 1C at 40 °C. The LE consisted of 1M NaPF_6_ (99%, Sigma-Aldrich) in the EC/DEC/FEC carbonate mixture. Na│NaPSiOM│P2-NMFT cells were cycled at 40 °C using a constant C rate of 0.1C after one activation cycle at 0.05C (1C = 263 mA g^−1^) between 2.0 and 4.0 V. Na│NaPSiOM│NFPP cells were cycled at 25 °C using a constant C rate of 1C (1C = 129 mA g^−1^) between 1.7 and 3.8 V. The contact pressure during pouch cell testing was ensured by two glass plates clamped on both sides of the cell. Finally, HC│NaPSiOM│NFPP sodium-ion cells were cycled at 0.1C (1C = 129 mA g^-1^) at 40 °C between 0.5 and 3.7 V, with an N/P ratio of 1.1. Cycling of the HC electrode in Na half-cells was carried out using a C rate of 0.1C (1C = 250 mA g^-1^) at 40 °C between 0 and 2.0 V. All the galvanostatic cycling measurements were performed using a Maccor 4000 battery tester.

*5 Ex situ Analyses*

*Ex situ* X-ray photoelectron spectroscopy (XPS) analyses were carried out on sodium-metal electrodes retrieved under argon atmosphere from Na║Na cells after 5 cycles at 50 µA cm^−2^ and 40 °C. The analyses were conducted using a Phoibos 150 XPS spectrometer equipped with a monochromatic Al-Kα (1486.6 eV) X-ray source. High-resolution scans were acquired at 200 W with a pass energy of 20 eV and a step-size of 0.1 eV in a fixed analyzer transmission mode. The depth profiling was performed with a focused Ar^+^ ion gun (5 keV) and an ion filter for 15 min (sputtering rate: 0.8 nm min^−1^). The calibration was conducted using the hydrocarbon reference peak at 285 eV. The spectra were fitted using Shirley background and Voight line shape.

*Ex situ* scanning electron microscopy (SEM) and energy dispersive X-ray spectroscopy (EDX) analyses were performed on sodium-metal electrodes retrieved under argon atmosphere from Na║NVP cells employing either NaPSiOM or the reference LE after 100 cycles carried out at 1C and 40 °C. Upon cell disassembly, the membrane did not show any adhesive behavior, allowing for the smooth separation from the sodium-metal anode. To ensure data integrity, SEM images were acquired on the central region of the anode surface, which remains unaffected by potential mechanical stress from the cell casing. SEM-EDX analyses were also performed on the NaPSiOM surface at the pristine state. All the SEM micrographs were acquired using a ZEISS Crossbeam XB340 microscope operated at 5 kV, and the corresponding EDX data were obtained with an Ultim Extreme™ (Oxford instruments) EDX spectrometer coupled to the SEM.

**Figure S1.** **(a)** FT-IR spectra of the of the NaPSiO ionomer as well as the precursors NaMTFSI and PMMS. **(b)** Magnification of the FT-IR spectra of NaPSiO and PMMS in the 2650 − 2450 cm^-1^ region.
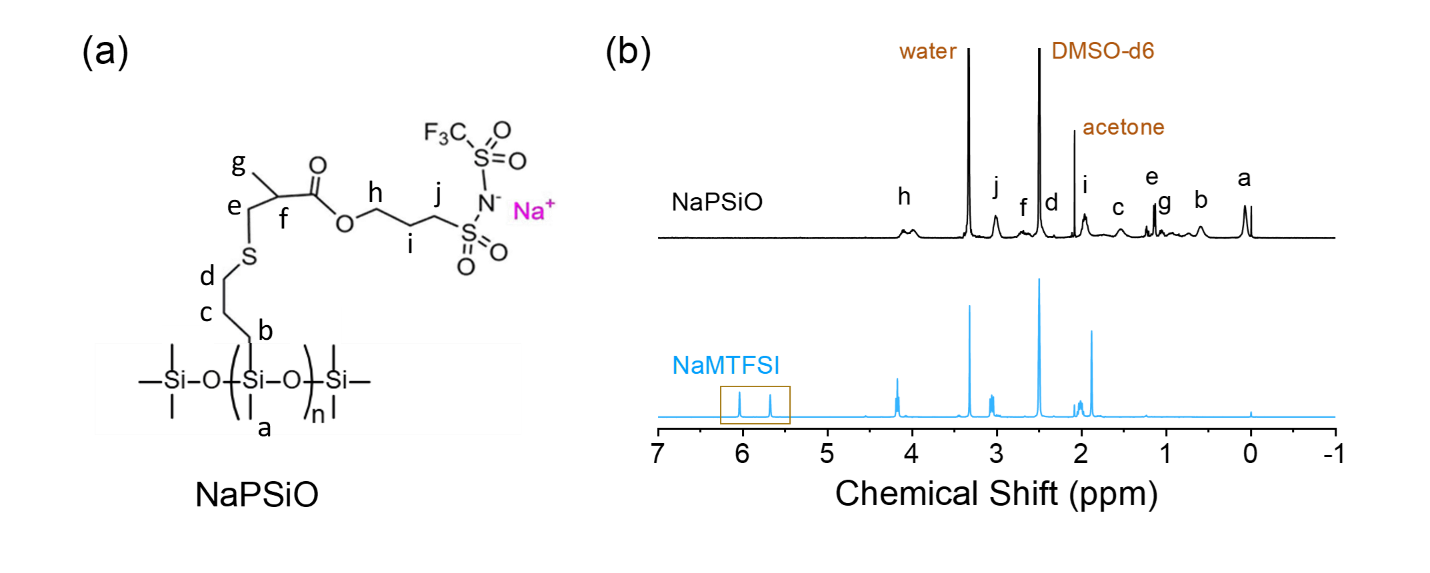


**Figure S2**. **(a)** Chemical structure of the NaPSiO ionomer with the corresponding H assignments for the ¹H NMR spectra presented in **(b)** along with the ¹H NMR spectra recorded for NaMTFSI for comparison; including a labeling of the peaks related to residual water (3.33 ppm), DMSO-d₆ (2.54 ppm), and acetone (2.09 ppm).


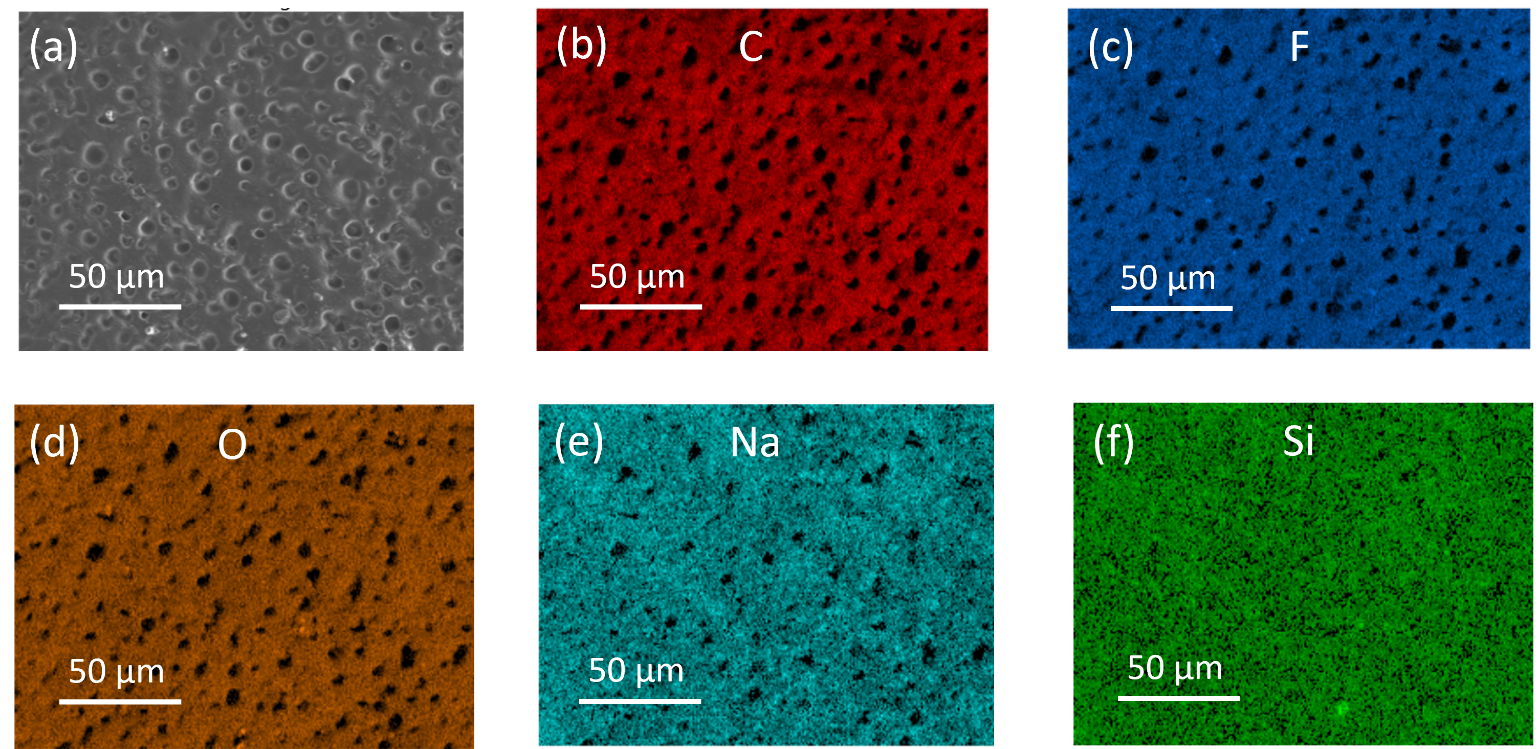


**Figure S3.** **(a)** SEM micrograph of the dry NaPSiOM and **(b–f)** the corresponding EDX elemental mapping of **(b)** C, **(c)** F, **(d)** O, **(e)** Na, and **(f)** Si.

**Figure S4**. Determination of the glass transition temperature *T_g_* by means of DSC for **(a)** carbonate-soaked NaPSiOM and **(b)** dry NaPSiOM.


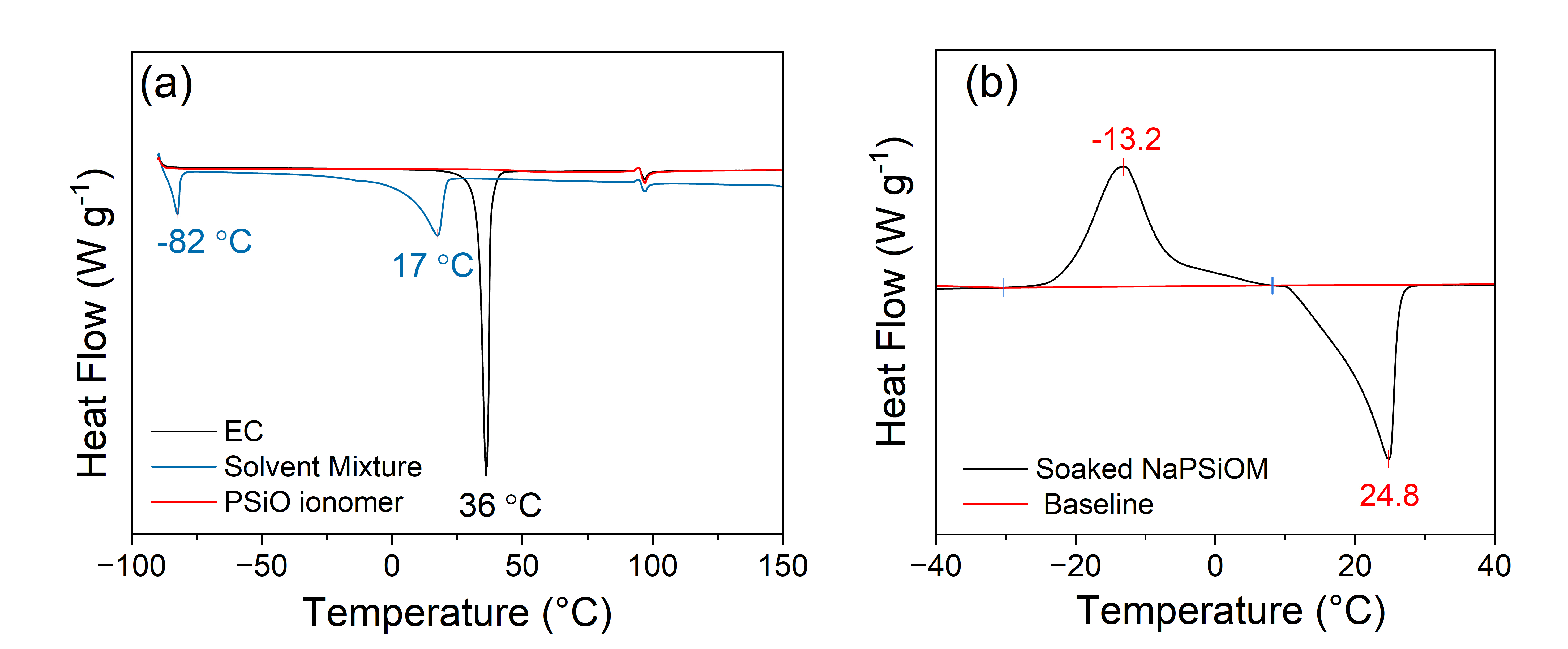


**Figure S5.** **(a)** DSC analysis of pure EC, the solvent mixture EC/DEC/FEC, and the NaPSiO ionomer recorded within a temperature range from -100 to 150 °C. **(b)** Peak analysis of the cold-crystallization peak and the subsequent melting peak extracted from the DSC data recorded for soaked NaPSiOM, as presented in **Figure 1d** (the given baseline is a user-defined linear interpolation of the heat flow signal, anchored at local minima in regions where no thermal transitions occur (approximately -30°C and +8°C), to isolate the latent heat of the transitions from the sample's heat capacity background).

Additional DSC measurements for pure EC and the solvent mixture used herein (incl. the data recorded for the dry NaPSiO ionomer serving as reference) show a melting peak at ca. 36 °C for pure EC, whereas the EC/DEC/FEC solvent mixture shows a depressed EC-related melting peak at ca. 17 °C and a second peak near −82 °C, which is assigned to DEC-rich domains (**Figure S5a**). These results are consistent with literature reports for an EC/DEC (1:1 v:v) system, where the EC-related melting temperature has been observed at 17-18 °C as well.^[5]^ For the soaked membrane (**Figure S5b**), the melting peak shifts to about 25 °C. The slightly increased EC-related melting temperature can be attributed to a microphase separation induced by NaPSiO and the PVdF-HFP polymer matrix.^[6]^ In fact, the observation of a cold crystallization at about −13 °C suggests a recrystallization under confinement of EC-rich domains, which frequently results in more stable and purer solvent crystals, which is in good agreement with the observed increase of the melting temperature compared with the NaPSiO-free solvent mixture (**Figure S5a**).

The enthalpy (*ΔH*) associated to the crystallization/melting peaks observed in **Figure S5b** was calculated by using **Equation S3**, and the results are reported in **Table S1**.

$\boldsymbol{\Delta H}=\frac{1}{\beta}\int_{T_{1}}^{T_{2}} \dot{q}_{norm}dT$ (S3)

where $\dot{q}$ is the heat flow in W g⁻¹ and *β* is the heating rate (0.167 °C S⁻¹).

**Table S1**. Enthalpy change (*ΔH*) associated to the peaks shown in **Figure S5b**, determined using **Equation S3**.

| **Temperature** | **ΔH** [J/g] |
| --- | --- |
| -13.2 °C | 19.2 |
| 24.8 °C | 23.4 |


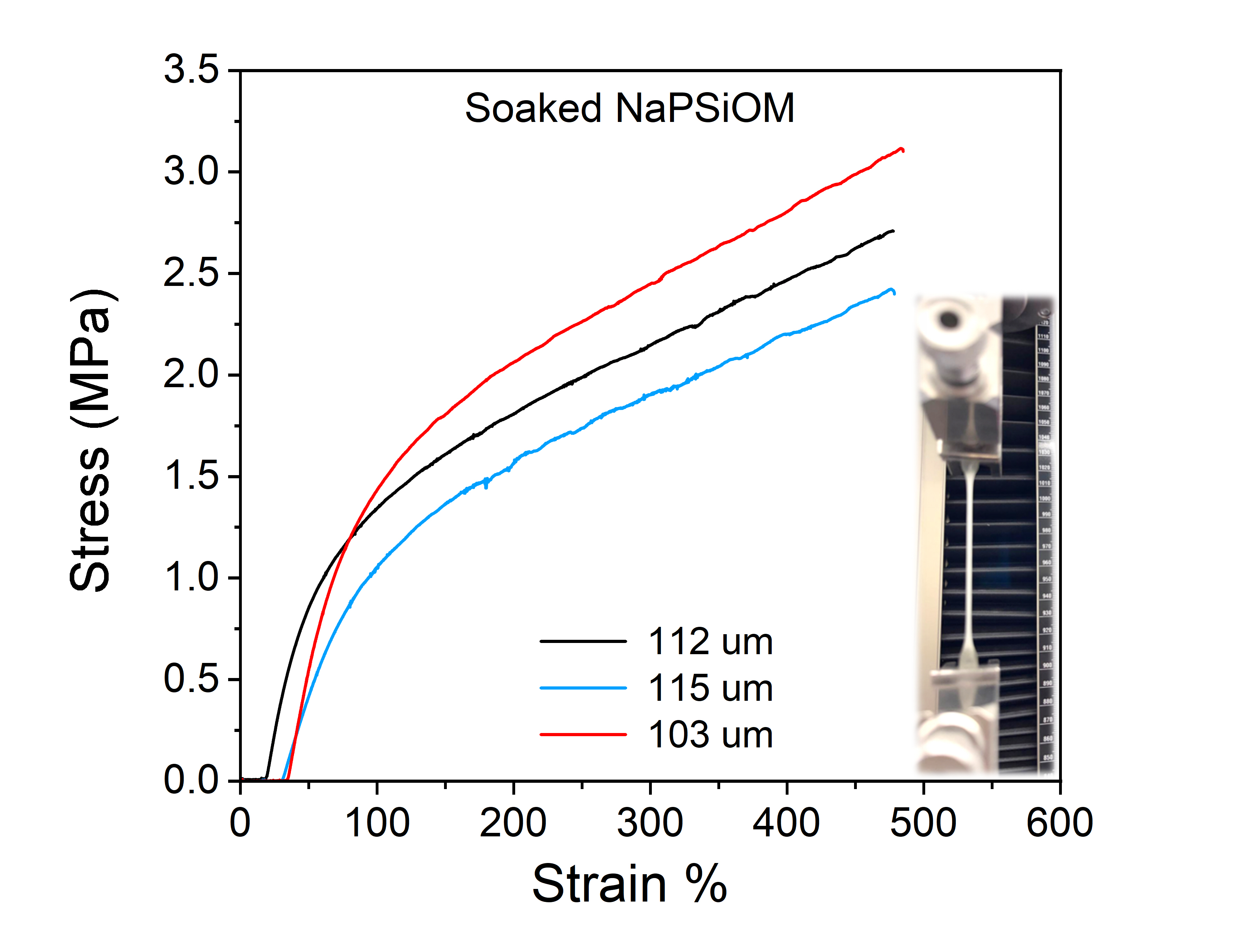


**Figure S6.** Stress–strain curves of soaked NaPSiOM membranes with different thicknesses (103, 112, and 115 μm). The inset shows a photograph of the membrane during the tensile test.

**Table S2**. Mechanical properties of soaked NaPSiOM membranes with different thicknesses subjected to tensile strength tests (**Figure S6**): the Young’s modulus (*Eₜ*), tensile strength (*σₘ*), and elongation at break (*εₘ*).

| **Thickness** [µm] | **E_t_** [MPa] | **σ_M_** [MPa] | **ε_M_** [%] |
| --- | --- | --- | --- |
| 112 | 0.032 | 2.77 | 477 |
| 115 | 0.048 | 2.41 | 476 |
| 103 | 0.059 | 3.16 | 483 |

**Figure S7**. Nyquist plots of the EIS data recorded for SS│NaPSiOM│SS cells for the determination of the ionic conductivity in the temperature range from 20 to 90 °C (see the NLLS fitting presented in **Table S3** and the corresponding Arrhenius-type plot in **Figure 2a**).

**Table S3**. NLLS fitting of the Nyquist plots shown in **Figure S7** recorded at various temperatures for symmetric SS│NaPSiOM│SS cells for the determination of the ionic conductivity (cf. the corresponding Arrhenius-type plot in **Figure 2a**).

| **Temperature** | ***R_b_*** [Ω] | **σ** [mS cm^−2^] |
| --- | --- | --- |
| 20 °C | 73.6 | 0.17 |
| 30 °C | 45.9 | 0.27 |
| 40 °C | 31.4 | 0.40 |
| 50 °C | 22.5 | 0.55 |
| 60 °C | 17.3 | 0.72 |
| 70 °C | 13.6 | 0.91 |
| 80 °C | 11.2 | 1.11 |
| 90 °C | 9.7 | 1.29 |


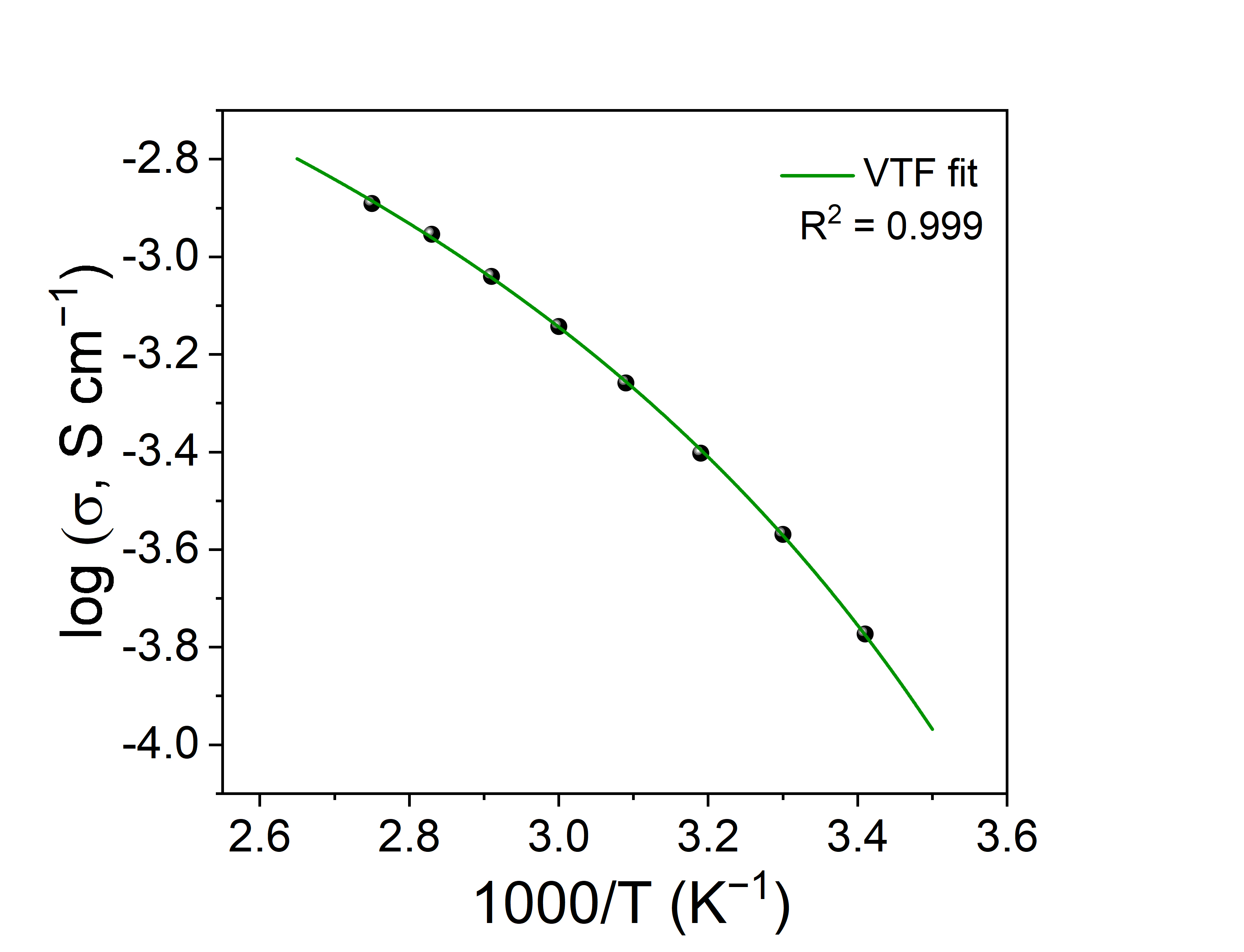


**Figure S8**. VTF fitting of the ionic conductivity data plotted as log(σ) vs. 1000/T (cf. the corresponding Arrhenius plot in **Figure 2a**).

$\sigma=A\exp\left( -\frac{E_{a}}{R\left( T-T_{0} \right)} \right)$ (S4)

**Table S4.** Parameters obtained from the VTF fitting of the ionic conductivity as shown in **Figure S8** using **Equation S4**. Note that the given value for the (temperature-dependent) activation energy $E_{a}$ reflects an average value across the investigated temperature range from 20 to 90 °C.

| **A** | **E_a_** [eV] | **T_0_** [k] | **R^2^** | **Reduced χ^2^** |
| --- | --- | --- | --- | --- |
| -1.966 | 0.293 | 210 | 0.999 | 7.6×10^−5^ |

**Figure S9**. Magnification of the FT-IR spectrum of the carbonate-soaked NaPSiOM, highlighting the C=O stretching region from 1900 to 1650 cm^-1^.

**Table S5**. Parameters used in **Equation S2** to calculate *t_Na+_* for NaPSiOM at 40 °C using the Watanabe method^[7,8]^ (cf. **Figure 2b**). The NLLS fitting of the Nyquist plot was carried out using the equivalent circuit *R_b_(R_1_Q_1_)(R_2_Q_2_)*, as also depicted in the inset in **Figure 2b**.

| **ΔV** [V] | ***I_ss_*** [A] | ***R_b_*** [Ω] | ***R_i_* (*R_1_* + *R_2_*)** [Ω] | ***t_Na+_*** |
| --- | --- | --- | --- | --- |
| 0.01 | 2.49×10^-6^ | 54 | 3951 | 0.82 |

**Figure S10**. Magnification of the LSV plots recorded for Na│NaPSiOM│SS cells upon oxidation in the region from 3.6 to 5.0 V at 20 and 40 °C with an indication of the electrochemical stability towards oxidation when setting the threshold for the evolving current density to 3 µA cm^-2^.


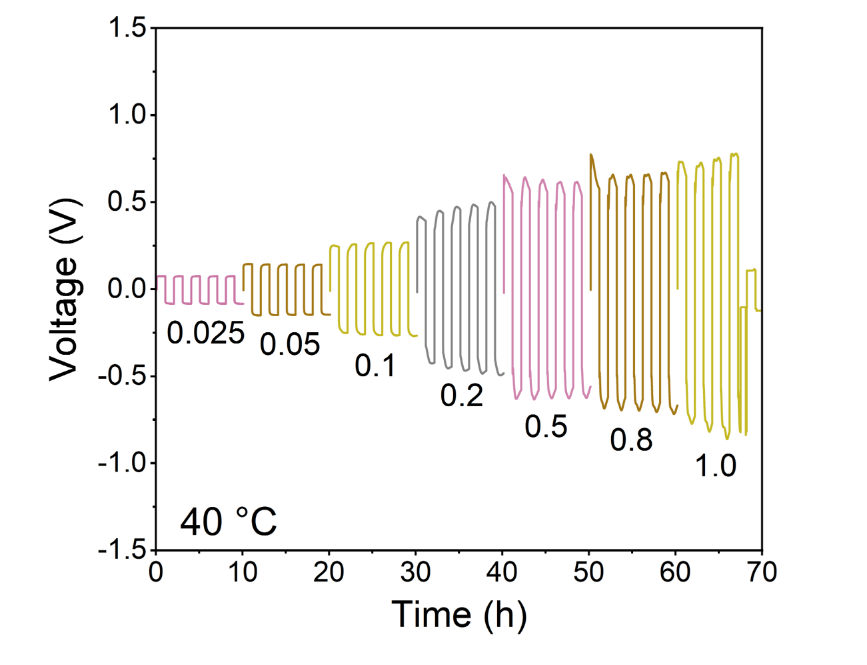


**Figure S11**. Determination of the critical current density (CCD) via galvanostatic stripping/plating conducted in Na│NaPSiOM│Na cells at 40 °C, applying a stepwise increasing current density from 0.025 to 1.0 mA cm⁻², with a step time of 1 h for each plating/stripping cycle.

**Figure S12**. Determination of the limiting current density for NaPSiOM at 40 °C by applying an increasing potential to symmetric Na║Na cells.

**Figure S13**. Magnification of the dis-/charge profiles recorded for Na│NaPSiOM│NVP cells at 0.5C and 40 °C (cut-off voltages: 2.4 and 3.9 V; cf. **Figure 4d)**.


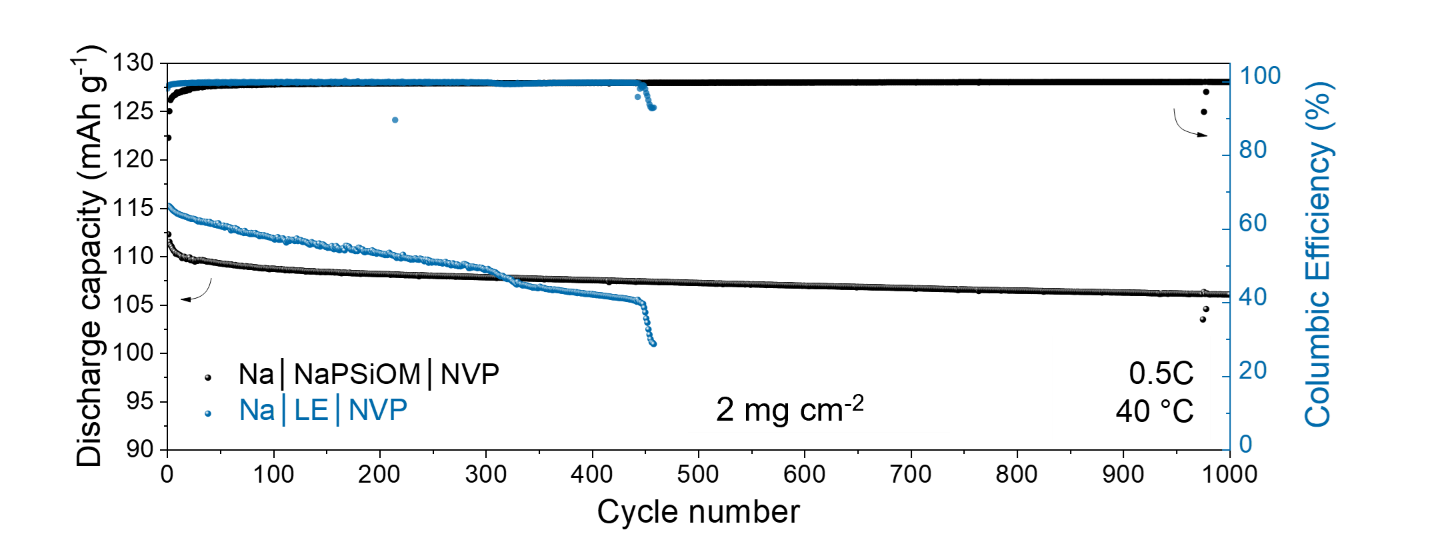


**Figure S14**. Comparison of the galvanostatic cycling of Na║NVP cells employing NaPSiOM or LE as the electrolyte (T = 40 °C; C rate: 0.5C; cut-off voltages: 2.4 and 3.9 V); the data related to NaPSiOM are also displayed in **Figure 4c**.


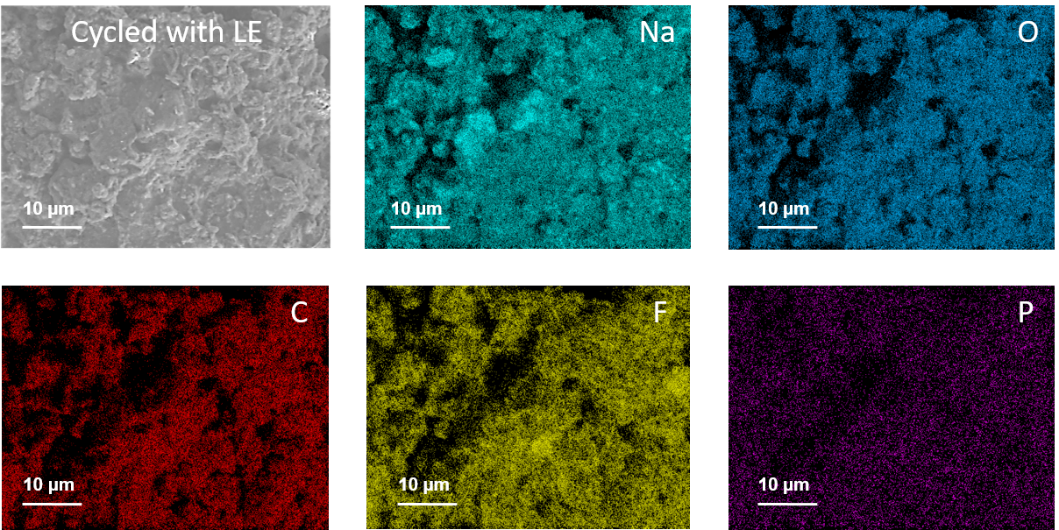


**Figure S15.** EDX elemental mapping of the surface of Na-metal anodes recovered after 100 cycles in Na│LE│NVP cells (cf. the corresponding SEM micrographs **Figure 5c,e,g**).

**Table S6.** EDX analysis of the elements Na, O, F, C and P, as detected on the Na-metal electrodes after 100 cycles in Na│LE│NVP cells, cycled at 1C between 2.4 and 3.9 V.

| **Element** | **wt.%** |
| --- | --- |
| Na | 39.0 |
| O | 24.4 |
| F | 17.9 |
| C | 13.1 |
| P | 5.6 |
| Total | 100 |


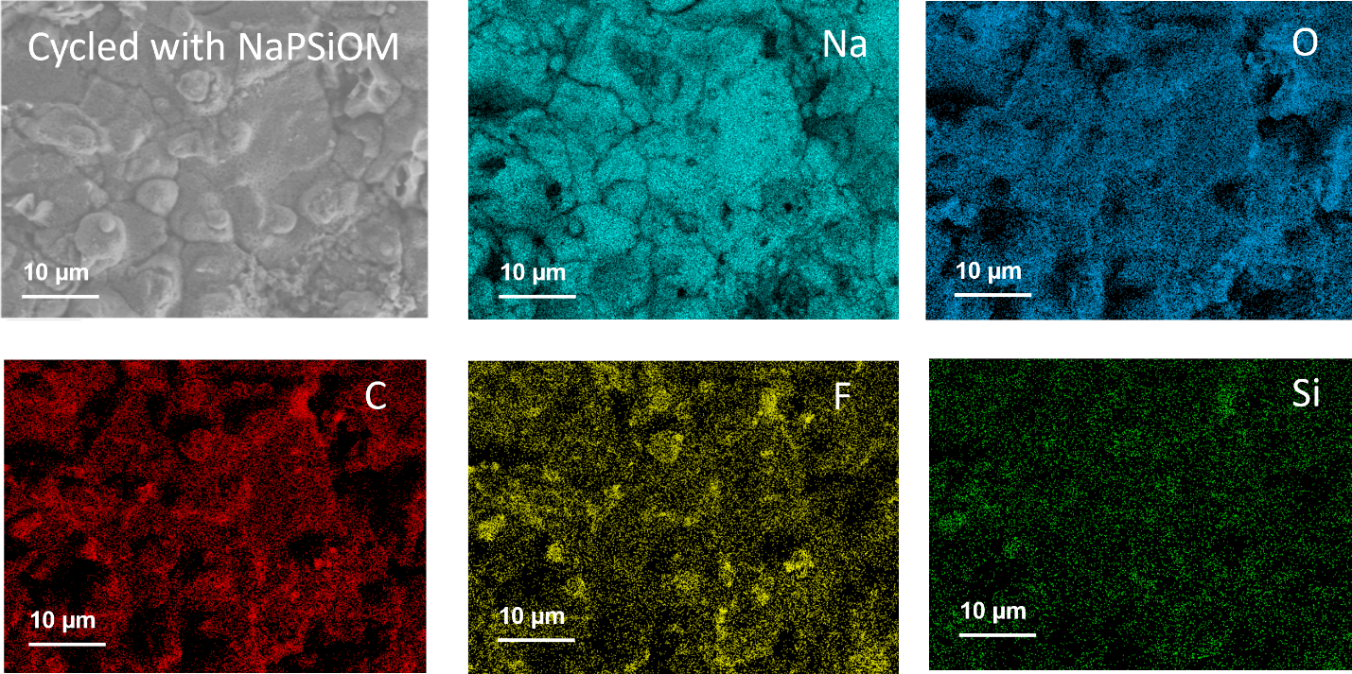


**Figure S16.** EDX elemental mapping of the surface of Na-metal anodes recovered after 100 cycles in Na│NaPSiOM│NVP cells (cf. the corresponding SEM micrographs **Figure 5b,d,f**).

**Table S7.** EDX analysis of the elements Na, O, C, S, F, and Si, as detected on the Na-metal electrodes after 100 cycles in Na│NaPSiOM│NVP cells, cycled at 1C between 2.4 and 3.9 V.

| **Element** | **wt.%** |
| --- | --- |
| Na | 69.1 |
| O | 14.5 |
| C | 8.4 |
| S | 3.8 |
| F | 3.1 |
| Si | 1.1 |
| Total | 100 |


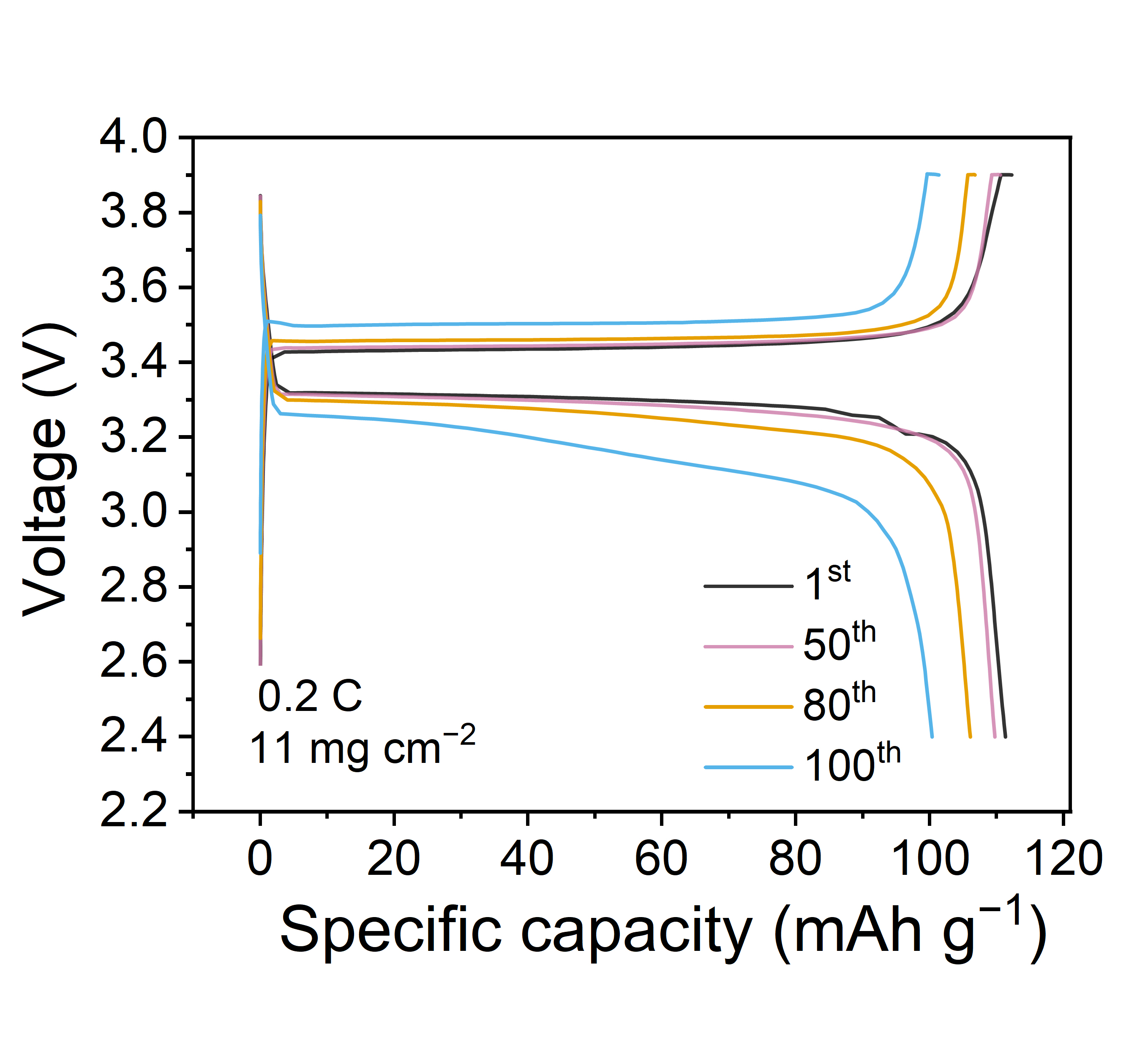


**Figure S17.** Selected galvanostatic dis-/charge profiles recorded for Na│NaPSiOM│NVP cells at 40 °C between 2.4 and 3.9 V using NVP electrodes with an active material mass loading of ca. 11 mg cm^-2^ and applying a C rate of 0.2C (cf. **Figure 7c**).


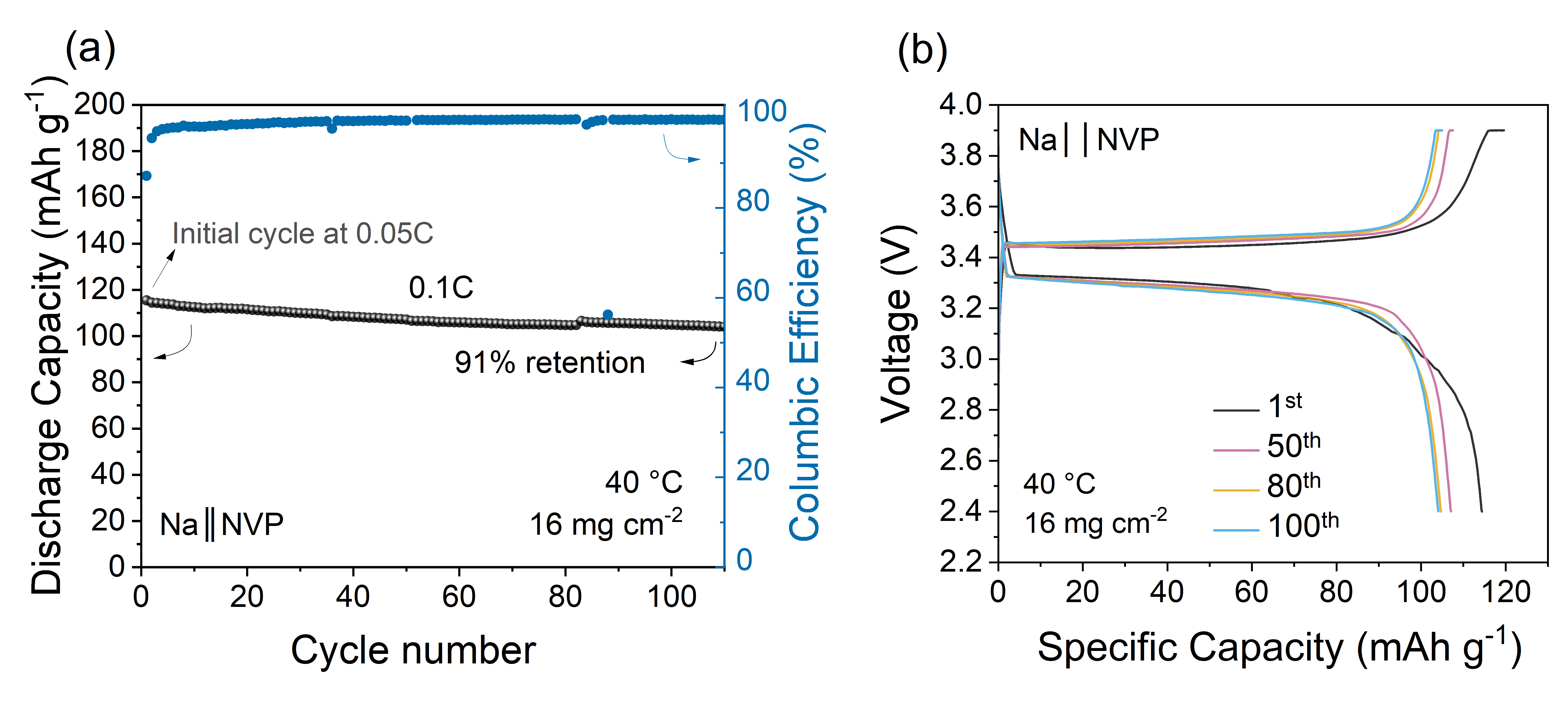


**Figure S18.**Selected dis-/charge profiles recorded for Na│NaPSiOM│NVP cells with an active material mass loading of ca. 16 mg cm^−2^ at 0.1C after one formation cycle at 0.05C (T = 40 °C; cut-off voltages: 2.4 and 3.9 V; 1C = 118 mA g^−1^; cf. **Figure 7d**).


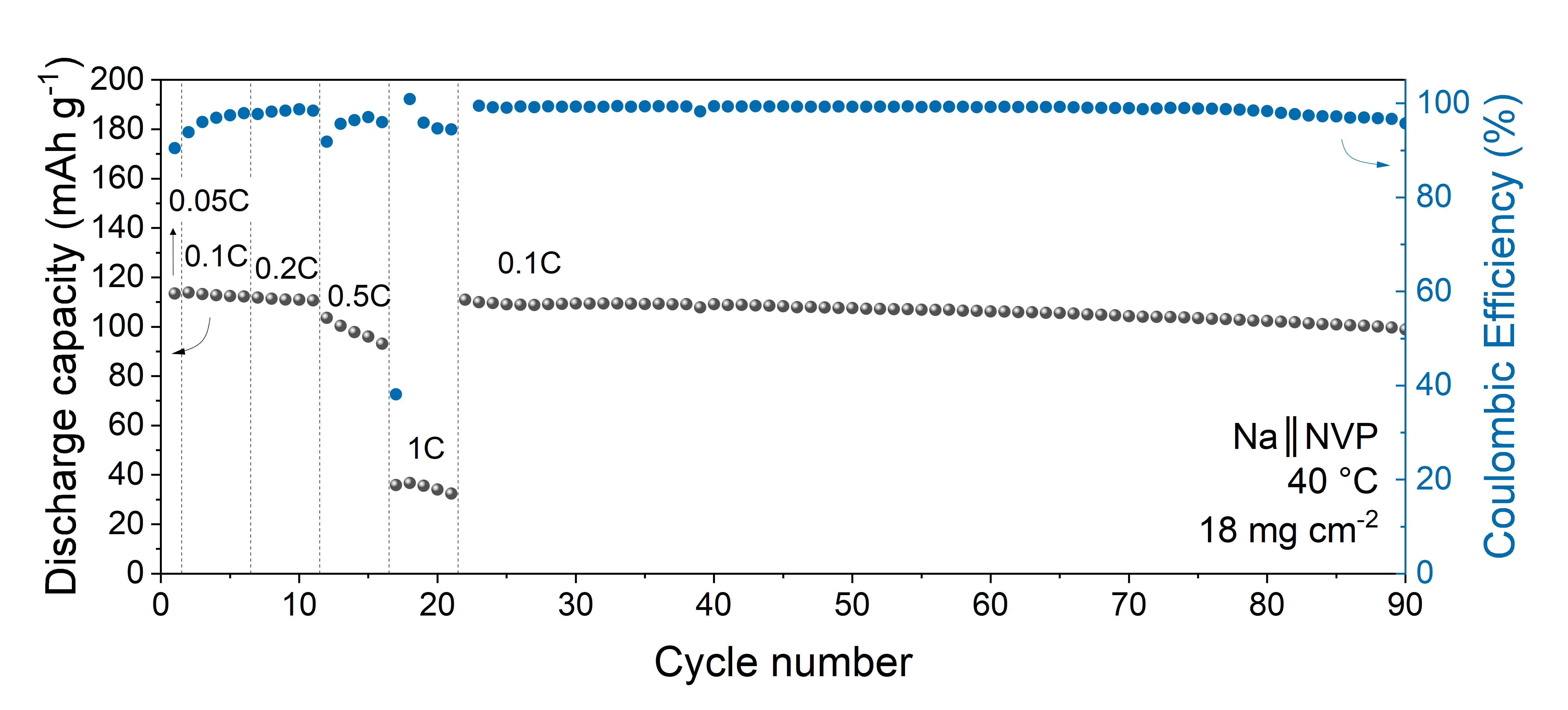


**Figure S19.** Galvanostatic cycling of Na│NaPSiOM│NVP cells with an active material mass loading of around 18 mg cm^−2^ at 40 °C at varying C rates after one formation cycle at 0.05C (cut-off voltages: 2.4 and 3.9 V; 1C = 118 mA g^−1^).


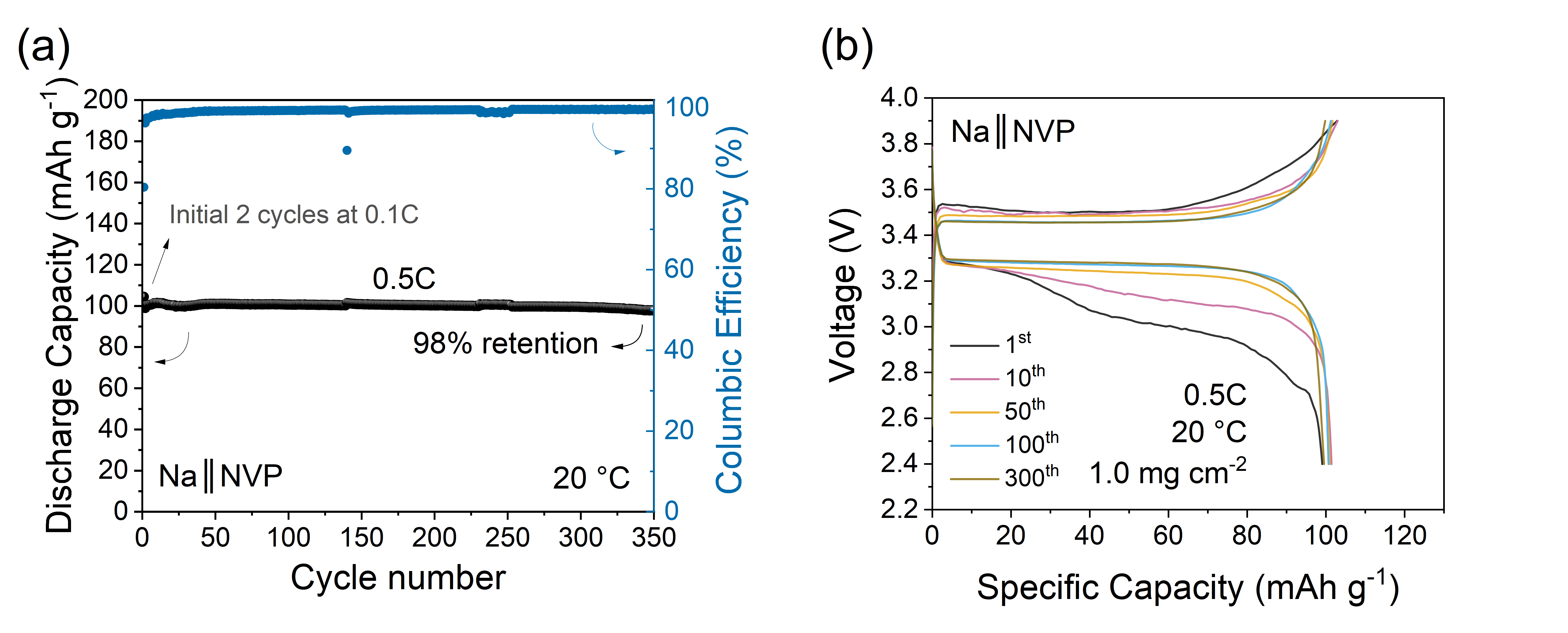


**Figure S20.** **(a)** Galvanostatic cycling of Na│NaPSiOM│NVP cells with an active material mass loading of about 1 mg cm^−2^ at 20 °C at 0.5C after two formation cycles at 0.1C (cut-off voltages: 2.4 and 3.9 V; 1C = 118 mA g^−1^) and **(b)** selected dis-/charge voltage profiles.

Interestingly, the first cycle discharge profile in **Figure S20b** exhibits an additional plateau, at around 3.0 V and a little below, which steadily increases in terms of average potential upon further cycling, as apparent from the 10^th^ discharge plateau, until there is only the characteristic single discharge plateau observed at around 3.3 V starting from the 50^th^ cycle. This behavior has been attributed earlier to the passivation layer formed on the sodium-metal counter electrode,^[9]^ and we may assume that we observe this only at 20 °C owing to the slower overall dynamics under these conditions.

**Table S8.** Comparison of the cells employing the NaPSiOM electrolyte, as reported herein, with the most representative works employing a Na^+^ single-ion polymer electrolytes (SIPEs) reported in the literature.

| Architecture of SIP Electrolytes | Cycling Performance  cc = coin cell; pc = pouch cell | | | | | | |
| --- | --- | --- | --- | --- | --- | --- | --- |
|  | *t_Na+_* | Test  T (°C) | Mass  Loading  (mg cm^-2^) | Current | Capacity (mAh g^−1^) | Capacity Retention | Cycle Number |
| NaPA/ PVdF-HFP ^[10]^ (EC/DMC swelled) | 0.83 | 20  **(cc)** | -- | 0.2C | 81.4 | ~74% | 50 |
| PSTB/PVCA PSP10-GPE ^[11]^ (EC/DEC) | 0.88 | 25  **(cc)** | -- | 0.5C | 85.5 | 98.6% | 200 |
| NaPTAB/ PVdF-HFP ^[12]^  (PC swelled) | 0.91 | 60  **(cc)** | ~1.56 | 0.5C | ~85 | 90% | 500 |
| NaBFMB/TMPT crosslinked NaSIE ^[13]^ | 0.91 | RT  **(cc)** | ~1.6 | 0.5C | 85.7 | 86.8% | 150 |
| PISC/ PVdF-HFP ^[14]^  (NaClO_4_ EC/PC) | 0.66/  0.75 | RT  **(cc)** | 1–1.5 | C/10 | 59 | 82.6% | 200 |
| Multi-block ionomer SIPE ^[15]^  (65 wt% EC) | 0.96 | 40  **(cc)** | 2.8 ± 0.1 | 0.2C | 107 | 84.7% | 1000 |
| NaSTFSI-co-PET-MP/4A / PVdF-HFP ^[16]^  (EC:DMC:FEC) | – | 40  **(cc)** | 1.3 ± 0.4 | C/10 | 147 | 98% | 40 |
| PEGMEM-co-SSS@ZIF-8 ^[17]^ | 0.87 | 80  **(cc)** | -- | 1C | ~75 | 96% | 300 |
| NaSDCM-PET-MP/4A- PVdF-HFP SIPE ^[18]^ | – | 40  **(cc)** | 1.3 ± 0.4 | 0.1C | 102 | – | 200 |
| NaMTFSI/diene/PETMP/ PVdF-HFP SIPE ^[19]^ | – | 40  **(cc)** | 1.3 ± 0.4 | C/10 | 119 | 68% | 200 |
| NaPSiO/PVdF-HFP  (EC/DEC/FEC)  *This work** | **0.82** | **40**  **(cc);**  **RT**  **(pc)** | **2-18 (cc);**  **5 (pc)** | **1C**  **(cc/pc)** | **108 (cc);**  **83 (pc)** | **92% (cc);**  **90% (pc);** | **1300 (cc);**  **600 (pc)** |


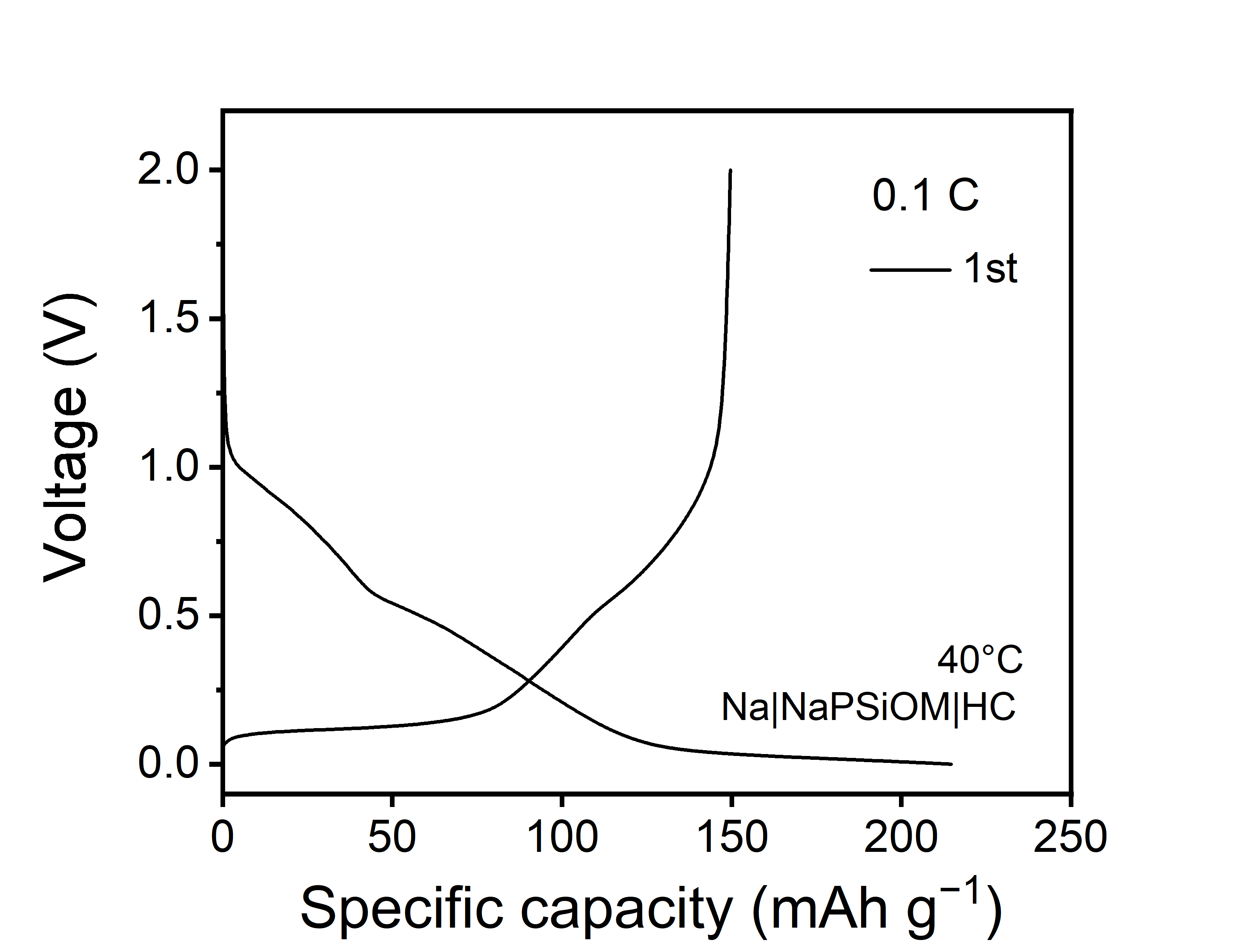


**Figure S21.** First dis-/charge profile recorded for Na│NaPSiOM│HC cells cycled at 0.1C (1C = 250 mA g^-1^) at 40 °C between 0 and 2.0 V.

**References**

1. H.-P. Liang, Z. Chen, X. Dong, et al., "Photo-Cross-Linked Single-Ion Conducting Polymer Electrolyte for Lithium-Metal Batteries," Macromolecular Rapid Communications 43, 12 (2022): 2100820. https://doi.org/10.1002/marc.202100820

2. L. Porcarelli, A.S. Shaplov, M. Salsamendi, et al., "Single-Ion Block Copoly(Ionic Liquid)S as Electrolytes for All-Solid State Lithium Batteries," ACS Applied Materials & Interfaces 8, 16 (2016): 10350. 10.1021/acsami.6b01973

3. A.S. Shaplov, P.S. Vlasov, M. Armand, et al., "Design and Synthesis of New Anionic “Polymeric Ionic Liquids” with High Charge Delocalization," Polymer Chemistry 2, 11 (2011): 2609. 10.1039/C1PY00282A

4. M. Patel, K. Mishra, N.A. Chaudhary, et al., "A Sodium Ion Conducting Gel Polymer Electrolyte with Counterbalance between 1-Ethyl-3-Methylimidazolium Tetrafluoroborate and Tetra Ethylene Glycol Dimethyl Ether for Electrochemical Applications," RSC Advances 14, 20 (2024): 14358. <https://doi.org/>10.1039/ D4RA01615G

5. M.S. Ding, K. Xu, S. Zhang, T.R. Jow, "Liquid/Solid Phase Diagrams of Binary Carbonates for Lithium Batteries Part Ii," Journal of the Electrochemical Society 148, 4 (2001): A299. 10.1149/1.1353568

6. S. Abbrent, J. Plestil, D. Hlavata, et al., "Crystallinity and Morphology of PVdF–HFP-Based Gel Electrolytes," Polymer 42, 4 (2001): 1407. https://doi.org/10.1016/S0032-3861(00)00517-6

7. M. Watanabe, S. Nagano, K. Sanui, N. Ogata, "Estimation of Li^+^ Transport Number in Polymer Electrolytes by the Combination of Complex Impedance and Potentiostatic Polarization Measurements," Solid State Ionics 28-30, (1988): 911. https://doi.org/10.1016/0167-2738(88)90303-7

8. L.-Y. Lin, C.-C. Chen, "Accurate Characterization of Transference Numbers in Electrolyte Systems," Journal of Power Sources 603, (2024): 234236. https://doi.org/10.1016/j.jpowsour.2024.234236

9. A. Rudola, D. Aurbach, P. Balaya, "A New Phenomenon in Sodium Batteries: Voltage Step Due to Solvent Interaction," Electrochemistry Communications 46, (2014): 56. https://doi.org/10.1016/j.elecom.2014.06.008

10. Q. Pan, Z. Li, W. Zhang, et al., "Single Ion Conducting Sodium Ion Batteries Enabled by a Sodium Ion Exchanged Poly(Bis(4-Carbonyl Benzene Sulfonyl)Imide-Co-2,5-Diamino Benzesulfonic Acid) Polymer Electrolyte," Solid State Ionics 300, (2017): 60. https://doi.org/10.1016/j.ssi.2016.12.001

11. P. Wang, H. Zhang, J. Chai, et al., "A Novel Single-Ion Conducting Gel Polymer Electrolyte Based on Polymeric Sodium Tartaric Acid Borate for Elevated-Temperature Sodium Metal Batteries," Solid State Ionics 337, (2019): 140. https://doi.org/10.1016/j.ssi.2019.04.022

12. L. Yang, Y. Jiang, X. Liang, et al., "Novel Sodium–Poly(Tartaric Acid)Borate-Based Single-Ion Conducting Polymer Electrolyte for Sodium–Metal Batteries," ACS Applied Energy Materials 3, 10 (2020): 10053. 10.1021/acsaem.0c01756

13. K. Liu, Y. Xie, Z. Yang, et al., "Design of a Single-Ion Conducting Polymer Electrolyte for Sodium-Ion Batteries," Journal of the Electrochemical Society 168, 12 (2021): 120543. 10.1149/1945-7111/ac42f2

14. S. Das, S. Jana, M. Orságh, et al., "Building Sodium Metal Battery with Polyisoprene-Based Air-Stable Single-Ion Gel Polymer Electrolyte," ACS Applied Energy Materials 6, 10 (2023): 5113. 10.1021/acsaem.3c00430

15. X. Dong, X. Liu, H. Li, S. Passerini, D. Bresser, "Single-Ion Conducting Polymer Electrolyte for Superior Sodium-Metal Batteries," Angewandte Chemie International Edition 62, 43 (2023): e202308699. https://doi.org/10.1002/anie.202308699

16. C. Wunder, T.-L. Lai, E. Šić, et al., "Sodium 4-Styrenesulfonyl(Trifluoromethanesulfonyl)Imide-Based Single-Ion Conducting Polymer Electrolyte Incorporating Molecular Transporters for Quasi-Solid-State Sodium Batteries," Journal of Materials Chemistry A 12, 32 (2024): 20935. 10.1039/D4TA02329C

17. J. Zhang, Y. Wang, Q. Xia, et al., "Confining Polymer Electrolyte in Mof for Safe and High-Performance All-Solid-State Sodium Metal Batteries," Angewandte Chemie International Edition 63, 16 (2024): e202318822. https://doi.org/10.1002/anie.202318822

18. C. Wunder, L. Graeber, D. Bresser, M. Zarrabeitia, S. Passerini, "Understanding the Component-Driven Influence on the Electrochemical Properties in Single-Ion Polymer Electrolytes for Sodium-Based Batteries," ACS Applied Polymer Materials 7, 8 (2025): 4895. 10.1021/acsapm.4c04234

19. C. Wunder, L. Hildebrand, L. Gräber, et al., "Influence of the Backbone Chemistry and Side-Chain Spacer Flexibility in Sodium Single-Ion Conducting Polymer Electrolyte for Sodium-Batteries," EES Batteries 2, 1 (2026): 282. 10.1039/D5EB00145E
